# Noninvasive theta-burst stimulation of the human striatum enhances striatal activity and motor skill learning

**DOI:** 10.1038/s41593-023-01457-7

**Published:** 2023-10-19

**Authors:** Maximilian J. Wessel, Elena Beanato, Traian Popa, Fabienne Windel, Pierre Vassiliadis, Pauline Menoud, Valeriia Beliaeva, Ines R. Violante, Hedjoudje Abderrahmane, Patrycja Dzialecka, Chang-Hyun Park, Pablo Maceira-Elvira, Takuya Morishita, Antonino M. Cassara, Melanie Steiner, Nir Grossman, Esra Neufeld, Friedhelm C. Hummel

**Affiliations:** 1 Defitech Chair of Clinical Neuroengineering, Neuro-X Institute and Brain Mind Institute, École Polytechnique Fédérale de Lausanne, Geneva, Switzerland; 2 Defitech Chair of Clinical Neuroengineering, Neuro-X Institute and Brain Mind Institute, Clinique Romande de Réadaptation, École Polytechnique Fédérale de Lausanne, Sion, Switzerland; 3 Department of Neurology, University Hospital Würzburg, Würzburg, Germany; 4 Institute of Neuroscience, Université Catholique de Louvain, Brussels, Belgium; 5 Department of Health Sciences and Technology, ETH Zurich, Zurich, Switzerland; 6 Neuroscience Center Zurich, Zurich, Switzerland; 7 School of Psychology, Faculty of Health and Medical Sciences, University of Surrey, Guildford, UK; 8 Department of Neuroradiology, Sion Hospital, Sion, Switzerland; 9 Department of Brain Sciences, Imperial College London, London, UK; 10 United Kingdom Dementia Research Institute, Imperial College London, London, UK; 11 Foundation for Research on Information Technologies in Society, Zurich, Switzerland; 12 Clinical Neuroscience, University of Geneva Medical School, Geneva, Switzerland

**Keywords:** Cognitive neuroscience, Cognitive ageing, Biological techniques, Neuroscience

## Abstract

The stimulation of deep brain structures has thus far only been possible with invasive methods. Transcranial electrical temporal interference stimulation (tTIS) is a novel, noninvasive technology that might overcome this limitation. The initial proof-of-concept was obtained through modeling, physics experiments and rodent models. Here we show successful noninvasive neuromodulation of the striatum via tTIS in humans using computational modeling, functional magnetic resonance imaging studies and behavioral evaluations. Theta-burst patterned striatal tTIS increased activity in the striatum and associated motor network. Furthermore, striatal tTIS enhanced motor performance, especially in healthy older participants as they have lower natural learning skills than younger subjects. These findings place tTIS as an exciting new method to target deep brain structures in humans noninvasively, thus enhancing our understanding of their functional role. Moreover, our results lay the groundwork for innovative, noninvasive treatment strategies for brain disorders in which deep striatal structures play key pathophysiological roles.

## Abstract (web summary)

The Hummel lab demonstrated that the striatum can be successfully and focally reached noninvasively via transcranial electrical temporal interference stimulation in humans, which resulted in improvements of motor learning in older adults.

## Main

Neuromodulation of cortical and subcortical brain structures is an important step toward improving our understanding of neuronal processing across brain networks, thereby allowing us to probe and decipher causal brain–behavior relationships^1^. Existing noninvasive brain stimulation (NIBS) techniques, including transcranial magnetic stimulation (TMS) and transcranial electric stimulation (tES), have been widely used to investigate healthy and pathological systems^2^. However, these approaches show a steep depth–focality tradeoff^3^, with focality decreasing as depth increases. As a result, deep brain structures, such as the basal ganglia and hippocampus, cannot be reached directly without diffusely costimulating the overlying cortex^3,4^. Thus, these deep structures have been accessible only through the use of invasive brain stimulation techniques^5^. To perform deep brain stimulation in healthy subjects and reduce the side effects associated with invasive procedures, new concepts and technologies are needed. One exciting possible solution was recently proposed by Grossman et al., who introduced the transcranial temporal interference stimulation (tTIS) technique in rodents^6^. During tTIS, two pairs of electrodes are placed on the head, with each pair delivering a high-frequency (HF) alternating current. Importantly, this frequency should be sufficiently high and thus not affect the mechanisms maintaining neuronal electrical homeostasis. Moreover, a small frequency shift is applied between the two alternating currents. The superposition of the electric fields creates an envelope oscillating at this low-frequency difference, which in turn influences neuronal activity. By optimizing the electrode placement and current intensity ratio across stimulation channels, the maximal amplitude of the envelope can be steered; hence, the primary focus of neuromodulation can be directed toward individual deep brain structures while minimizing neuromodulation in the surrounding and/or overlying areas^6^.

In the present work, we employed the tTIS strategy in humans to study the effects of striatal neuromodulation on local and network brain activity and associated motor learning behavior. Motor learning is a crucial process for a variety of daily life activities, ranging from learning to use tools to playing musical instruments and recovering from motor disabilities, and has been the focus of numerous neuroscientific studies in recent decades^7,8^. These works have revealed that multiple deep brain structures play essential roles in motor learning and motor control, with the striatum being a key hub in this motor network^7,9^. However, in human neuroscience, the contribution of these structures has been largely assessed via associative methods, for example, through indirect inferences from neuroimaging results. In particular, two main motor learning phases have been identified: an initial fast phase, during which subjects substantially increase their performance by integrating sensory inputs, and a later slower phase, during which improvements are less pronounced and are gained slower^10^. Neuronal substrates are recruited depending on the ongoing phase. The striatum is one of the central nodes and essentially involved in both phases of learning^11,12^. The activation and engagement of its substructures dynamically change throughout the learning process, with the caudate nucleus implicated during the initial fast learning phase and the putamen more associated with the slower phase^12,13^. Even within the putamen, different compartments have been found to change their activity over time, that is, the activation shifts from the associative (rostrodorsal) part to the sensorimotor (caudoventral) part during training^14^. It should be noted that the duration of the learning phases is highly task-specific, for example, the fast phase could last approximately 30 min for simple laboratory-based motor tasks, such as those used in the present work, or up to several months for complex everyday activities, such as learning a piece of music^10,15^.

A critical limitation of existing human neuroimaging techniques, for example, functional magnetic resonance imaging (fMRI), positron emission tomography and electroencephalography (EEG), is that these approaches provide only associative evidence of the brain–behavior relationships underlying motor learning^1,16^. Most causal evidence originates from animal work^17,18^, striatal lesion studies of patient cohorts^19^, or invasive deep brain stimulation studies of connected nuclei^20,21^, which have indicated the significant role of the striatum in motor learning. However, since human data have been obtained from patients with altered network properties due to disorder-related neurodegeneration or lesions, we cannot draw comprehensive conclusions on the physiology of healthy systems. The noninvasive modulation of striatal activity during motor training with the tTIS strategy may allow us to address this critical gap.

In this article, we applied tTIS to the striatum in randomized, double-blind, crossover designs, demonstrating the possibility of noninvasively targeting the striatum in humans without coactivating overlying cortices beneath the electrodes. Moreover, we characterized local and network effects on brain activity using fMRI recordings during stimulation (experiment 1) and quantified behavioral effects by studying the evolution and efficiency of acquiring novel hand-based motor skills in healthy young and healthy older subjects (experiment 2).

## Results

### Experiment 1

#### tTIS modulates motor task-induced striatal activity

In experiment 1, task-based fMRI was acquired during a sequential finger tapping task (SFTT)^15,22^ with concomitant theta-burst patterned tTIS or HF control stimulation. Theta-burst patterned stimulation was chosen as the active condition because this form of stimulation has been shown to induce long-term potentiation (LTP)-like effects in previous animal and human works^23,24^. Specifically, a train of 2-s theta-bursts was delivered every 10 s to mimic an intermittent theta-burst stimulation protocol^24^. The task was divided into six blocks, with each block consisting of ten 30-s repetitions of the SFTT with concomitant stimulation alternated with 30 s of rest without stimulation.

**Fig. 1:**
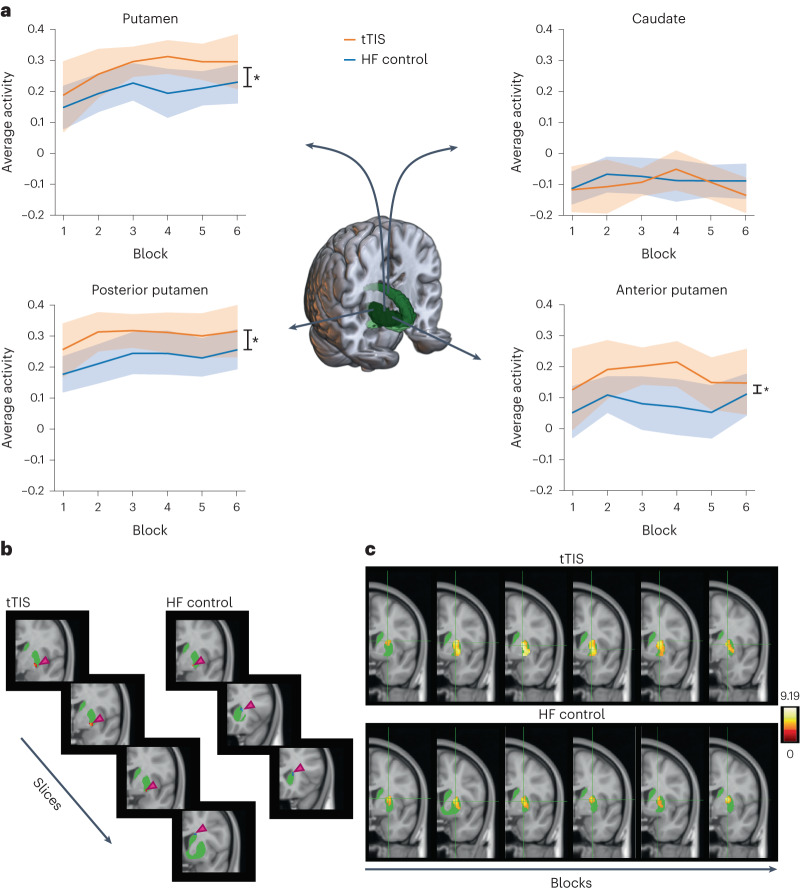
Results of the task-based fMRI experiment—local activity. **a**, Average BOLD activity in the putamen (top left) and caudate (top right) during tTIS and HF control stimulation (*n* = 13, one influential point removed based on Cook’s distance). tTIS led to significantly higher activity in the putamen (two-sided pairwise comparisons via estimated marginal means: *t*(276) = −2.55, *P* = 0.01, *d* = −0.41, Tukey adjustment) but not in the caudate. The average BOLD activity in the posterior (bottom left) and anterior (bottom right) putamen during tTIS and HF control stimulation was also studied (*n* = 14). tTIS led to significantly higher activity in both areas (one-sided ANOVA with Satterthwaite’s approximations: *F*(1, 299) = 13.47, *P* = 0.0003, p*η*^2^ = 0.04 (small)). The lines indicate the measure of center (mean value across the stimulation condition) and the shaded areas represent standard errors (SEs). **b**, Voxels showing a trend of linear changes (a one-sided *t* contrast, uncorrected *P* = 0.01 at the voxel level, and uncorrected at the cluster level) over time in the striatum during tTIS are shown on the left and during HF control stimulation on the right, on a group level. The sections are ordered from caudal to rostral. Hot colors represent increased activity over time, while cold colors represent decreased activity. The green area indicates the striatum. **c**, Qualitative characterization of the location of the activity during each of the six blocks during tTIS (top) and HF control stimulation (bottom). Data are shown on a group level, highlighting voxels involved in the task when compared with baseline (a one-sided *t* contrast, uncorrected *P* = 0.001 at the voxel level, and uncorrected at the cluster level). The left side corresponds to the early training phase, and the right side corresponds to the later training phase. The shift from the superior to the inferior striatum is consistent with previous observations^14,25^. The green area indicates the striatum. The color bar indicates the *t* statistic.

To investigate the effects of the stimulation on the target region, the average activity in subregions of the striatum was extracted, as shown in Fig. 1a. A significant effect of the region (*F*(1, 276) = 260.01, *P* = 1.14 × 10^−41^, partial eta-squared (p*η*^2^) = 0.49 (large)) and a significant region *x* stimulation interaction (*F*(1, 276) = 4.48, *P* = 0.035, p*η*^2^ = 0.02 (small)) were detected. The region effect can be explained by higher activity in the putamen than in the caudate during the task (*t*(276) = −16.13, *P* = 1.14 × 10^−41^, *d* = −1.83, Tukey adjustment). The interaction effect can be explained by higher activity in the putamen during tTIS than during HF control stimulation (*t*(276) = −2.55, *P* = 0.01, *d* = −0.41, Tukey adjustment), while no difference was observed in the caudate region (*t*(276) = 0.45, *P* = 0.65, *d* = 0.07, Tukey adjustment). This result suggests that LTP-like plasticity effects induced via tTIS preferentially increased activity in a striatal subregion (putamen) that was more activated during the motor task. To better understand the effect of stimulation within the putamen, we distinguished the anterior and posterior parts of the putamen (Fig. 1a, bottom). The stimulation effect was confirmed, which is consistent with the results reported above (*F*(1, 299) = 13.47, *P* = 0.0003, p*η*^2^ = 0.04 (small)), with tTIS leading to increased activity. This increase was not specific to a particular part of the putamen and was present in both subregions, as no significant region × stimulation interaction was observed (*F*(1, 299) = 0.13, *P* = 0.72, p*η*^2^ = 0.0004 (micro)). Finally, a significant subregion effect was also observed (*F*(1, 299) = 37.03, *P* = 3.56 × 10^−9^, p*η*^*2*^ = 0.11 (medium)), with the posterior part of the putamen showing higher activation than the anterior part (*t*(299) = −6.09, *P* = 3.56 × 10^−9^, *d* = −0.66, Tukey adjustment).

Next, we characterized the temporal changes in activity within the striatum during learning based on previous findings^14,25^. Areas in the right striatum showing a trend of linear increases or decreases in activation over time were extracted for each of the two stimulation conditions (uncorrected *P* = 0.01 at the voxel level, and uncorrected at the cluster level). Figure 1b shows that, for both stimulation conditions, the activity in the lower part of the putamen increased, while the activity in the superior part of the caudate decreased, which is consistent with the literature^14,25^. The evolution of these functional changes over time is visualized in Fig. 1c, in which the striatal activity and peak locations are depicted. During tTIS, a greater part of the sensorimotor striatum is involved over time, with activity observed also in the inferior part of the striatum. This shift was less pronounced when HF control stimulation was applied during the task, with activity still located between the superior and inferior parts of the striatum during the last block of training.

In brief, the analyses indicate that simultaneous application of theta-burst patterned tTIS and motor training could induce a differential effect on activity in striatal subregions and accelerate the shift of activation toward sensorimotor subregions, which has been linked with learning in prior studies^14,25^. This finding suggests that tTIS can regulate learning phase-dependent recruitment patterns in the target region.

#### Striatal tTIS modulates activity in the motor network

**Fig. 2:**
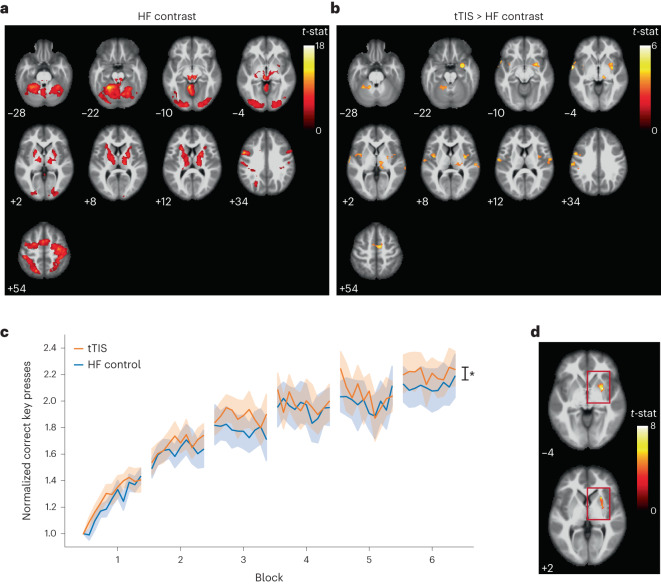
Results of the task-based fMRI experiment—network activity. **a**, BOLD activity during the motor task with concomitant HF control stimulation. The regions in the motor network involved in the SFTT are shown. Significant clusters are shown for an one-sided *t* contrast, uncorrected *P* = 0.001 at the voxel level, and FDR-corrected *P* = 0.05 at the cluster level. **b**, Comparison of BOLD activity between tTIS and HF control stimulation. Hot colors represent higher activity during tTIS. Significant clusters are shown for an one-sided *t* contrast, uncorrected *P* = 0.001 at the voxel level, and FDR-corrected *P* = 0.05 at the cluster level. **c**, Behavioral results of experiment 1 (*n* = 13, one influential point removed based on Cook’s distance). Performance is shown as the correct number of key presses normalized to the baseline. A significant effect of the stimulation was present, with tTIS leading to overall higher performance (one-sided ANOVA with Satterthwaite’s approximations: *F*(1, 1,560) = 6.35, *P* = 0.01, p*η*^2^ = 0.004 (micro)). The lines indicate the measure of center (mean value across the stimulation condition), and the shaded areas represent standard errors (SEs). **d**, Areas in the right striatum where activity was significantly modulated by the behavioral score (correct key presses) during tTIS. Significant clusters are shown for an one-sided *t* contrast, uncorrected *P* = 0.001 at the voxel level, and FDR-corrected *P* = 0.05 at the cluster level. No significant clusters were observed during HF control stimulation.

**Fig. 3:**
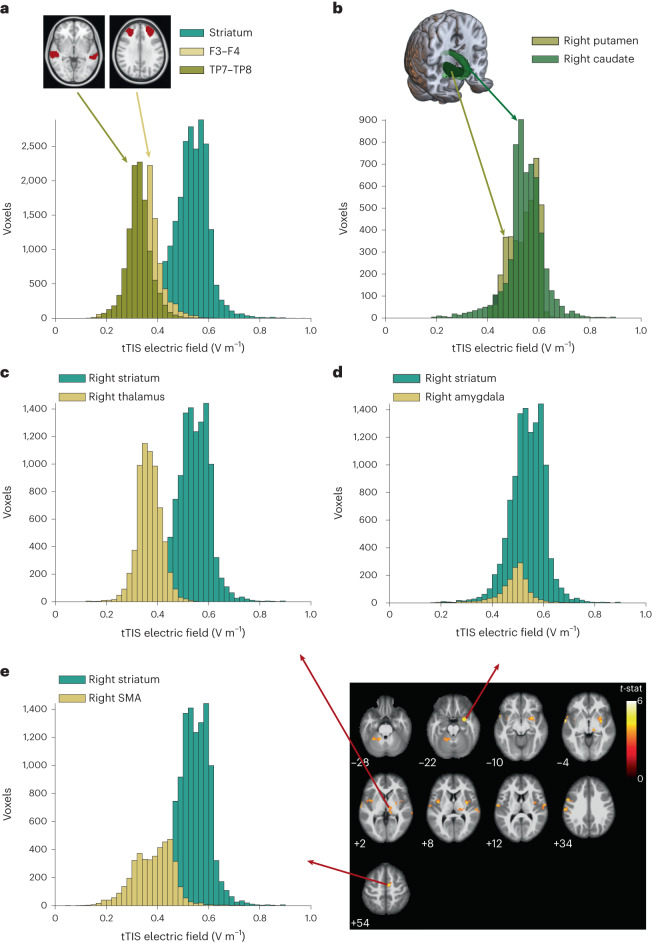
tTIS exposure strength in control regions with respect to the targeted striatum. Histogram depicting the tTIS exposure distribution within specific ROIs computed for a 2 mA current intensity per channel (peak to baseline). **a**, tTIS exposure distribution of voxels in 10-mm-radius spheres underneath the four stimulating electrodes, averaged for the frontal and posterior electrodes, compared with that in the bilateral striatum (putamen, caudate and nucleus accumbens). The horizontal axis scale was limited to the range [0, 1] for visualization purposes. As a result, nine values greater than 1 V m^−1^ were omitted, which most likely represented noise values at the edges of the brain mask. **b**, tTIS exposure distribution of voxels in subparts of the target region, namely the right putamen and right caudate. **c**–**e**, tTIS exposure distribution of voxels in supratentorial hubs showing stronger BOLD activation during the task-based fMRI experiment with concurrent tTIS than during HF control stimulation compared with that in the right striatum (putamen, caudate and nucleus accumbens). **c**, tTIS exposure distribution of voxels in the right striatum compared with voxels in the specific BNA^27^ regions of the thalamus, which contained voxels showing higher BOLD activity during the task-based fMRI experiment with concurrent tTIS than during HF control stimulation. **d**, tTIS exposure distribution of voxels in the right striatum compared with voxels in the specific BNA^27^ regions of the amygdala, which contained voxels showing higher BOLD activity during the task-based fMRI experiment with concurrent tTIS than during HF control stimulation. **e**, tTIS exposure distribution of voxels in the right striatum compared with voxels in the specific BNA^27^ regions of the SMA, which contained voxels showing higher BOLD activity during the task-based fMRI experiment with concurrent tTIS than during HF control stimulation.

To evaluate how the modulatory effects of striatal theta-burst patterned tTIS influence the rest of the brain, whole brain blood oxygen level-dependent (BOLD) activation during the motor task was compared between the tTIS and HF control stimulation. First, we characterized the regions involved in the motor task during HF control stimulation (Fig. 2a), which included the main nodes of the motor learning network, as expected; for review, please see, for example, Hardwick and colleagues^7^. Then, clusters with significantly higher activation during tTIS than during HF control stimulation were identified (uncorrected *P* = 0.001 at the voxel level, and false discovery rate (FDR)-corrected *P* = 0.05 at the cluster level) (Fig. 2b). Significantly higher activity was found in regions associated with the motor learning network for tasks performed with the left hand, including the right striatum (31.9% of the amygdala cluster), right thalamus and supplementary motor area (SMA), and left cerebellum (for the complete list of regions, see Supplementary Table 1). To evaluate whether these changes could be driven by striatal modulation, we performed a connectivity analysis (generalized psychophysiological interaction) from the right putamen to the motor regions showing higher activity during tTIS, which included the following areas: the clusters touching the cerebellum, thalamus and SMA. A significant effect of the cluster (*F*(2, 450) = 30.70, *P* = 3.18 × 10^−13^, p*η*^2^ = 0.12 (medium)) and a significant interaction stimulation × cluster (*F*(2, 450) = 3.26, *P* = 0.04, p*η*^2^ = 0.01 (small)) were obtained (Supplementary Fig. 1). The interaction was driven by a lowering of putamen–cerebellar connectivity induced by tTIS. One possible explanation is that the reduced connectivity interfered with the natural inhibitory influence of the putamen on the cerebellum^26^, leading to the observed increased BOLD activity in the cerebellum. In a further supplemental analysis, we also compared the estimated exposure strength to the tTIS field in supratentorial hubs with higher activity during tTIS to that of the striatal target region. The results indicated higher exposure levels in the striatum, as shown in Fig. 3. As a further step, we performed an electrophysiological control experiment in which we measured corticospinal excitability linked to the motor cortex with TMS, see Supplementary Fig. 2. The data suggest that neither tTIS nor HF control modulated corticospinal excitability linked to the motor cortex (Supplementary Table 2, stimulation × timing: *F*(2,34.03) = 1.19, *P* = 0.32, p*η*^2^ = 0.07 (medium)). These results strongly suggest that striatal tTIS successfully modulated activity in the striatum and the associated motor learning network without engagement of the overlaying cortices beneath the electrodes or the motor cortex with respect to the activity during the nonmodulating HF control stimulation.

Furthermore, we examined whether the BOLD signals below the electrodes were modulated by the stimulation condition. BOLD signals were extracted from 10-mm-radius spheres and the region of the Brainnetome atlas (BNA^27^) beneath the electrode location. The following regions in the BNA atlas were selected: the left and right A9/46d (dorsal area 9/46) underlying F3 and F4, respectively, and the left and right anterior superior temporal sulcus underlying TP7 and TP8, respectively. In these control regions, no effect of stimulation was found (*F*(1, 651) = 2.04, *P* = 0.15, p*η*^2^ = 0.003 (micro) when using the sphere model and *F*(1, 651) = 0.38, *P* = 0.54, p*η*^2^ = 0.0006 (micro) when investigating activity in the BNA regions); for additional details, please see Supplementary Fig. 3. These results strongly suggest that striatal tTIS successfully modulated activity in the striatum and the associated motor learning network without engagement of the overlaying cortices beneath the electrodes with respect to the activity during the nonmodulating HF control stimulation.

#### Striatal tTIS facilitates motor performance during learning

We next evaluated whether the neural effects of striatal tTIS were associated with changes in motor learning behavior by measuring changes in correct key presses during training (Fig. 2c). Significant effects of block (*F*(6, 1,560) = 243.22, *P* = 1.44 × 10^−219^, p*η*^2^ = 0.48 (large)) and stimulation (*F*(1, 1,560) = 6.35, *P* = 0.01, p*η*^2^ = 0.004 (micro)) were found. The significant block factor confirms the presence of learning effects during the task. The small but significant difference between the stimulation conditions highlights that, compared with HF control stimulation, motor task performance improved when tTIS was applied. The block × stimulation interaction was not significant, indicating that stimulation effects did not differ over time. Moreover, we investigated behavioral changes by computing the gain as the difference between the first and last task repetition of a session, the micro-online and the micro-offline learning. No significant effect of stimulation was found on the three measures (gain: *t*(13) = 0.23, *P* = 0.82, *d* = 0.06; for micro-online and offline learning, see Supplementary Fig. 4). According to these findings, we investigated whether the magnitude of BOLD activation in the striatal target region was related to behavioral outcomes. We considered the normalized number of correct key presses as a parametric modulator in the general linear model at the individual subject level. Group statistics restricted to the right striatum revealed significant modulation of striatal activity in the putamen, both in the anterior and posterior part, during tTIS (Fig. 2d and Supplementary Table 3). In contrast, activity was not significantly modulated by behavioral performance when HF control stimulation was applied. Together with the connectivity findings, this result strengthens the hypothesis of direct modulation of striatal activity via tTIS, which not only leads to higher activation but also supports a relationship between brain activity and behavior.

#### Absence of striatal tTIS effects without task-evoked activity

Next, we assessed whether striatal tTIS modulated BOLD signals in the absence of task-evoked activity. Resting-state fMRI (rs-fMRI) data were acquired in separate sessions. We analyzed seed-based connectivity using the right striatum as a seed. No interaction between stimulation conditions and the period (pre, during and post) was found. Additionally, we did not observe a difference in connectivity between striatal tTIS and HF control stimulation (uncorrected *P* = 0.001 at the voxel level, and FDR-corrected *P* = 0.05 at the cluster level). The absence of significant effects supports the hypothesis that behavioral coactivation is necessary to induce LTP-like plasticity effects via theta-burst patterned tTIS. Thus tTIS probably acts in a similar way as other conventional low-intensity plasticity-modulating tES protocols^28^.

### Experiment 2

#### Striatal tTIS effects are larger in older adults

In a second experiment, we validated the striatal theta-burst patterned tTIS approach in a behavioral experiment by recruiting a cohort of older adults (*N* = 15, right-handed, 9 females, mean ± standard deviation (s.d.) age 66.00 ± 4.61 years), who often demonstrate diminished performance gains in motor learning tasks^29,30^, have underlying brain networks that are less tuned^31,32^ and may have higher sensitivity to plasticity-modulating NIBS protocols^29,33^. Additionally, a new cohort of young healthy control subjects (*N* = 15, right-handed, 8 females, average age 26.67 ± 4.27 years) was recruited.

The subjects performed the SFTT in a shorter training session with longer blocks (seven 90-s blocks) while simultaneously receiving either striatal tTIS or HF control stimulation following a randomized, double-blind, crossover design. The overall duration of the training and stimulation was approximately three times shorter than that in experiment 1 to homogenize the protocol with previous behavioral studies^22,29^ and adapt it for follow-up investigations recruiting patient cohorts.

**Fig. 4:**
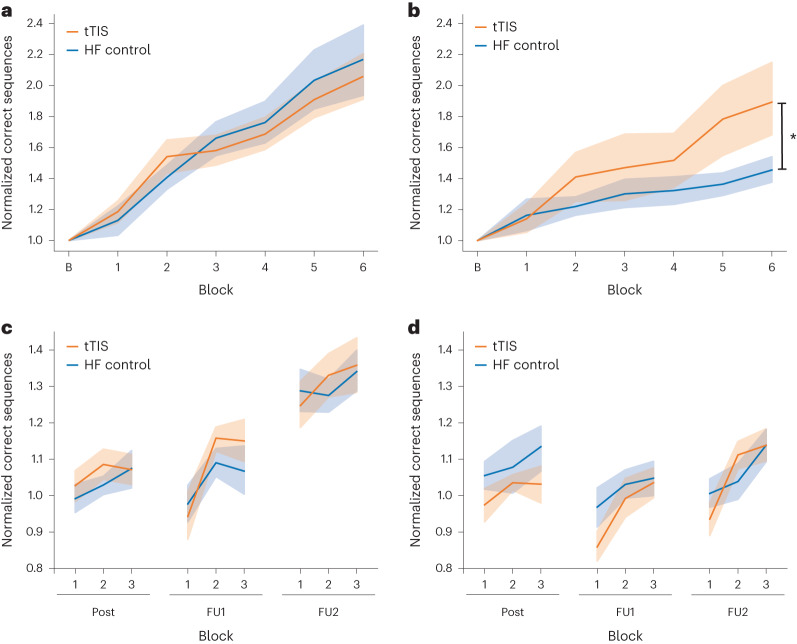
Behavioral experiment results in experiment 2. **a**, Motor task performance for the younger cohort (*n* = 14, one influential point removed based on Cook’s distance). Performance is shown as the correct number of sequences normalized to the baseline. No differences across stimulation conditions were observed. **b**, Motor task performance for the older cohort (*n* = 15). Performance is shown as the correct number of sequences normalized to the baseline. The post hoc analysis showed that this cohort performed significantly better during tTIS than HF control stimulation (two-sided pairwise comparisons via estimated marginal means: *t*(351) = 3.26, *P* = 0.001, *d* = 0.45, Tukey adjustment). **c**, Motor task performance during the follow-up (FU) sessions for the younger cohort (*n* = 15). Performance is shown as the correct number of sequences normalized to the last block of training. No differences across stimulation conditions were observed. **d**, Motor task performance during the follow-up sessions of the older cohort (*n* = 14, one influential point removed based on Cook’s distance). Performance is shown as the correct number of sequences normalized to the last block of training. No differences across stimulation conditions were observed. In **a**–**d**, the lines indicate the measure of center (mean value across the stimulation condition) and the shaded areas represent standard errors (SEs).

The analysis of the training phase indicated significant effects of block (*F*(6, 351) = 30.16, *P* = 3.78 × 10^−29^, p*η*^2^ = 0.34 (large)) and population (*F*(1, 27) = 4.36, *P* = 0.046, p*η*^2^ = 0.14 (large)), as well as significant stimulation × population (*F*(1, 351) = 6.71, *P* = 0.01, p*η*^2^ = 0.02 (small)) and block × population interaction effects (*F*(6, 351) = 2.29, *P* = 0.04, p*η*^2^ = 0.04 (small)) (Fig. 4a,b). The significant block × population interaction effect points toward differential learning dynamics across populations (for details, see Supplementary Table 4). Post hoc analysis of the stimulation × population interaction indicated a significant difference across stimulation conditions in the older cohort, with this cohort performing better during striatal tTIS than during HF control stimulation (*t*(351) = 3.26, *P* = 0.001, *d* = 0.45, Tukey adjustment). No significant difference was found in the younger cohort (*t*(351) = −0.45, *P* = 0.65, *d* = −0.06, Tukey adjustment). Moreover, we investigated behavioral changes by computing the gain as the difference between the first and last task blocks. A significant difference was found for the older cohort, with striatal tTIS leading to significantly higher gains than HF control stimulation (*V* = 96, *P* = 0.041, *d* = 0.76). No significant difference was found for the younger cohort (*V* = 40, *P* = 0.28, *d* = −0.39). The stimulation-associated effect in the younger and older cohorts was specific to the trained motor sequence, as no effects on motor performance were detected in an intermingled block, in which the order of key presses followed a predefined pseudorandom sequence (younger subjects: V = 53, *P* = 0.72, *d* = −0.19; older subjects: *V* = 82, *P* = 0.23, *d* = 0.44); for additional details, see Supplementary Fig. 5e,f. Additionally, we extracted micro-online or micro-offline learning to assess whether stimulation was acting specifically on one process or another. No significant effect of stimulation was found (for more information, see Supplementary Fig. 6). Because of the difference in results between the two young cohorts from experiment 1 and experiment 2, we merged the two datasets by separating the 90-s block of experiment 2 into three 30-s blocks and extracting the correct number of key presses to obtain a comparable dataset as in experiment 1. From the linear mixed model with stimulation and block as main factors, a significant effect of the two was found (stimulation: *F*(1, 999) = 3.88, *P* = 0.049, p*η*^2^ = 0.004; block*: F*(18, 999) = 48.29, *P* = 3.63 × 10^−122^, p*η*^2^ = 0.47). This is in line with the previously reported results showing stimulation and block effects, but no interactions. Hence, by considering all young subjects, tTIS still induced higher motor performance with respect to HF control stimulation. These results support the presence of a stimulation effect even when participants were performing a shorter training paradigm.

In both cohorts, the effect of stimulation on the follow-up measurements (post training (post), 90-min follow-up (FU1) and 24-hour follow-up (FU2)) was also investigated by analyzing performance normalized to the last block of the training (Fig. 4c,d). No significant stimulation effect was found (stimulation: *F*(1, 537) = 0.15, *P* = 0.70, p*η*^2^ = 0.0003 (micro); stimulation × follow-up: *F*(3, 537) = 0.22, *P* = 0.88, p*η*^2^ = 0.001 (micro); stimulation × population: *F*(1, 537) = 2.71, *P* = 0.10, p*η*^2^ = 0.005 (micro)).

#### Stimulation-associated sensations and control variables

The analyses of stimulation-associated sensations across stimulation conditions did not reveal differences in strength across the tested current intensity levels (0.5, 1.0, 1.5 and 2 mA per stimulation channel) (stimulation condition × current strength interaction, *F*(3, 837.95) = 0.06, *P* = 0.98, p*η*^2^ = 0.0002 (micro)) or sensation categories (stimulation condition × sensation category, *F*(6, 727.26*)* = 0.73*, P* = 0.63, p*η*^2^ = 0.006 (micro)); for additional details, see Supplementary Fig. 7. At the end of the experiment, the subjects correctly identified the session in which tTIS was applied at approximately chance level (experiment 1: task-based fMRI *P* = 0.75, rs-fMRI *P* = 0.55; experiment 2: *P* = 1.00 for both cohorts). This finding suggests the excellent blinding integrity of tTIS. Furthermore, the stimulation and time did not alter the subjects’ attention (experiment 1: *V* = 45, *P* = 0.66; experiment 2—young: *t*(14) = −0.54, *P* = 0.60; experiment 2—older: *V* = 35, *P* = 0.48) or fatigue levels (experiment 1: *t*(13) = *−*0.77, *P* = 0.46; experiment 2—young: *t*(14) *=* −1.02, *P* = 0.32; experiment 2—older: *t*(14) = *−*1.55, *P* = 0.14), as quantified with visual analog scales (VASs); for more information, see Supplementary Fig. 8.

## Discussion

The present study demonstrates for the first time that theta-burst patterned striatal tTIS can noninvasively modulate striatal activity and improve motor learning in humans. Specifically, striatal tTIS enhanced activity in the putamen with respective changes in core hubs of the associated brain network. Furthermore, striatal tTIS led to behavioral effects by increasing training gains during a motor learning task. The behavioral effect was particularly pronounced in older participants, who are known for their lower motor learning performance^29,30^ and less well-tuned underlying brain networks than younger participants^31,32^. In this work, we demonstrated that tTIS can overcome the depth–focality tradeoff observed in conventional NIBS techniques in humans, leading to sufficient current strengths to induce specific, focal and functionally relevant modulation of brain activity in deep brain structures, such as the striatum.

An important feature of tTIS is that it operates in the subthreshold range and does not directly induce neuronal action potentials. Thus, to further shape its topographic specificity, behavioral coactivation is probably needed. This argument is supported by the finding that striatal tTIS did not modulate seed-based functional connectivity in the target region during resting state; functional connectivity was quantified by concurrent rs-fMRI recordings. In other words, when the target region is at rest, tTIS alone cannot affect its connectivity. Furthermore, whole-brain analyses of task-evoked fMRI activity indicated tTIS-associated increases in functional activation only in the right striatum (Fig. 2b), which is strongly engaged in motor learning paradigms performed by the contralateral left hand^14,34^. This result is consistent with previous theories and experimental data acquired with brain slice-based electrophysiology, which suggests that the generation of neuroplasticity through low-intensity tES protocols requires coactivation by synaptic input or task-induced activity^35,36^. In addition, the presence of endogenous activity has been shown to lower the entrainment threshold of neuronal activity at certain resonant frequencies^37,38^.

How are these brain activation patterns induced by striatal theta-burst patterned tTIS linked to the associated behavioral enhancements? The present data suggest multiple, potentially complementary phenomena. First, the correlation analysis in the right striatum suggests that the magnitude of the tTIS-induced BOLD activity in the right putamen is associated with behavioral performance (Fig. 2d). Thus, stronger tTIS-associated activation in this specific target subregion is beneficial for supporting motor learning behavior. Second, the region of interest (ROI)-based analysis indicates that tTIS increased activity in the putamen, which accounts for a large part of the sensorimotor subdomain of the striatum (Fig. 1a)^39^, which has been linked to a more advanced stage of motor learning^14,25^. These stimulation-induced effects on activity were not observed in the caudate nucleus, which is linked to the associative subdomain (Fig. 1a)^39^. An additional hint is provided by the qualitative observation of the learning-phase-dependent activity shift toward inferior sensorimotor subregions over time (Fig. 1b,c), which appeared to be more pronounced when tTIS was applied with respect to the control condition. Overall, these findings suggest that striatal tTIS increases activity in striatal subregions linked to advanced learning phases^14,25^, thereby enhancing associated behavior. This observation is in line with data from animal models showing that LTP-induction protocols increase the BOLD signal in the target region and that population spikes and the slope of the excitatory postsynaptic potentials are positively correlated with the detected BOLD signal change^40^. It should be noted that the duration of the fast and slow learning phases is highly task-specific. Based on previous work using similar tasks^15^, both phases would be captured during the fMRI experiment (experiment 1; training duration including ~65 min).

**Fig. 5:**
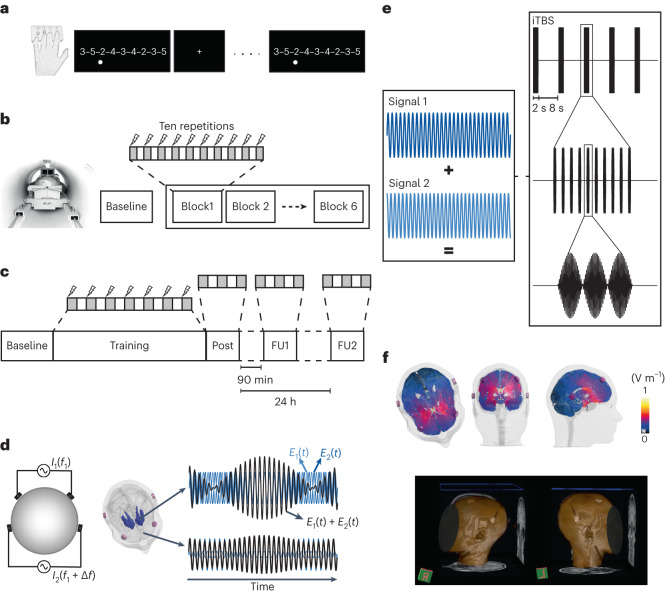
Experimental setup. **a**, Illustration of the SFTT^15,22^: participants were asked to reproduce a sequence displayed on a screen with a four-button box. **b**, Protocol of the task sessions in experiment 1: the baseline measurement was followed by training with concomitant stimulation, consisting of six blocks containing ten 30-s task repetitions alternated with 30-s rest periods. **c**, Protocol of experiment 2: the baseline measurement was followed by training with concomitant stimulation, consisting of seven repetitions of 1 min 30 s task blocks. A post assessment (Post) with three block repetitions was performed immediately after and in a follow-up assessment ~90 min (FU1) after and ~24 h (FU2) after the end of the stimulation. **d**, TI stimulation concept. On the left, two pairs of electrodes are shown on a head model, and currents are applied with frequencies of *f*_1_ and *f*_1_ + Δ*f*. On the right, the interference between the two electric fields within the brain is plotted at two different locations with high and low envelope modulation. **e**, In the pulsed stimulation mode, the frequency shift is only introduced during an exposure phase, for example, a burst. This allows the delivery of patterned intermittent theta-burst protocols in which three pulses of amplitude modulation at 100 Hz (burst phase) are repeated every 200 ms (theta frequency) for a 2-s train. The trains are repeated every 10 s. **f**, Electric field modeling with the striatal montage. Top: the electric tTIS exposure distribution in three chosen slices passing through the target region. Bottom: a 3D reconstruction of the structural MRI data, highlighting the electrode positioning.

Does striatal tTIS achieve focused neuromodulation with minimal exposure in the overlying cortices or other functionally relevant hubs of the motor learning network? The present results highlight stronger activation in typical core areas of the task-related motor network^7^ during tTIS. Even though this could be due to off-target stimulation, the likelihood is low on the basis of the tTIS field modeling results, which indicate lower exposure in these hubs than in the striatum, as shown in Fig. 3. Moreover, we note that similar results could have been found by stimulating cortical areas. However, there are several arguments against this assumption. First, the conducted electromagnetic simulations indicated that the estimated tTIS exposure strength is lower in BNA regions beneath the electrodes than in the striatum (Figs. 3 and 5f). Second, the present TMS control experiment revealed that theta-burst patterned tTIS does not modulate corticospinal excitability, as documented by similar patterned TMS or low-intensity tES protocols directly targeting the motor cortex^24,41^. Third, these results were confirmed by dedicated control analyses, indicating that striatal tTIS, which is capable of modulating BOLD activity patterns within the target structure, does not modulate BOLD signals in regions below the electrodes (Supplementary Fig. 3). These points argue against a relevant role of off-target stimulation of superficial cortical areas in mediating the reported effects on BOLD signals and behavior. The other possible component of the stimulation, namely, the HF control fields, was comparable in the tTIS and HF control stimulation; thus, this component should lead to similar effects in the brains of participants in both conditions, which is not consistent with the differences in the observed BOLD signal. Finally, taken together, the behavioral modulation and connectivity analysis between the putamen and motor regions support the hypothesis that the increased activity of the motor network hubs is more likely explained by striatal modulation. Our findings support a causal relationship between tTIS-induced changes in striatal activity and the connected areas of the motor network, in which activity was enhanced as a function of striatal stimulation in the absence of a substantial stimulation of the overlaying brain regions.

Does the tTIS effect depend on the stimulation dose or the lifespan stage? The present findings indicate the possible presence of a dose-dependent effect of striatal tTIS on behavior. In younger subjects, motor performance increased when the stimulation was applied for up to half an hour (in experiment 1), which is three times the amount of stimulation applied in experiment 2, during which no stimulation effects were observed. This difference could be due to an already optimal integration of task-relevant information, which is mainly important during early learning stages. The stimulation may thus support the optimization process of motor sequences during later stages of online learning via the cortico-basal ganglia loop^42^. Despite the shorter protocol, when investigating the striatal tTIS effects in an older population, the motor performance during training was better during tTIS than during HF control stimulation. Striatal theta-burst patterned tTIS led to strongly enhanced learning effects, with a 33.6% improvement over the control condition, even with this short training protocol. The pronounced response to the present intervention in the older participants can be explained by several potential reasons. One simple explanation is that healthy older adults have more room for improvement with striatal stimulation than young adults since healthy older adults show decreased motor learning abilities^29,30^ (Supplementary Fig. 9). Another possible explanation is based on previous imaging studies, which suggest suboptimal processing across dedicated brain networks during motor learning in older adults^43,44^. Thus, striatal tTIS might improve processing in this striato-cortical network and lead to corresponding behavioral improvements. Moreover, aging is related to structural and functional neurodegeneration and reduced brain plasticity, which are in turn associated with functional impairment^45,46^. Improving brain plasticity in regions affected by aging-related changes might result in the restoration of ‘natural’ dynamics, ultimately leading to behavioral improvements^29^. In line with this hypothesis, previous studies on neurological disorders also found stronger NIBS effects in patients showing stronger dysfunction and impairment^47^. Thus, the observed results suggest that striatal theta-burst patterned tTIS might have larger behavioral effects in cohorts with more pronounced brain malfunctions, such as in healthy older individuals and patients with brain lesions or neurodegenerative disorders. However, this hypothesis has not yet been tested.

What are the possible underlying mechanisms of striatal tTIS? In recent decades, several studies have consistently demonstrated that theta-burst patterned protocols can induce LTP-like plasticity effects^48^. Early evidence of modulatory effects originated from work conducted in brain slices, in which LTP was observed when two HF bursts were applied with interpulse intervals between 200 ms and 2 s (ref. ^23^). The first burst was hypothesized to act as a primer of postsynaptic activity. Hence, by manipulating stimuli timing, either LTP or long-term depression (LTD) can be induced on the basis of the postsynaptic state. The invention of patterned TMS enabled LTP-/LTD-like protocols to be applied in in vivo studies on monkey and human subjects. In this case, theta-burst patterned protocols demonstrated an ability to modulate cortical brain activity and plasticity in an LTP-/LTD-like manner^24,49^. Here we applied tTIS to achieve comparable LTP-like plasticity effects in the striatum, with the aim of supporting task-related synaptic plasticity. This approach is different from that taken in studies with conventional nonpatterned tTIS, which is assumed to achieve its effects through neural entrainment to the constant stimulation frequency. After the introduction of the temporal interference (TI) concept in the brain stimulation field by Grossman et al.^6^, the findings were reproduced in animal and computational models^50,51^; however, further investigations of the underlying mechanisms led to several hypotheses, open questions and disagreements between mechanistic models and experimentally observed responses^52^. For instance, experimental findings suggest that the stimulation effects depend on the time constants of the axon membrane and slow GABAergic inhibition (GABA_b_-type)^52^. This finding indicates selective responsiveness depending on neuron types and properties^53^. GABAergic receptors, including GABA_b_-type receptors, are highly expressed in the striatum, with evidence pointing toward the expression of GABA_b_ receptors on dopaminergic neurons^54^, which are important for the occurrence of striatal LTP effects^55^. Although LTP/LTD-like plasticity effects have been mainly studied in hippocampal and cortical slices, there is strong evidence that comparable phenomena occur in the basal ganglia. Previous work has shown that theta-burst patterned stimulation can induce LTP- and LTD-like effects in the dominant striatal cell type, GABAergic projecting medium spiny neurons, especially in cortical-striatal and thalamo-striatal inputs^56^. The results of the present study can thus be interpreted as demonstrating the ability of striatal tTIS to induce LTP-like plasticity effects and optimize the integration of cortical and thalamic inputs in the striatum. Currently, we speculate that this effect impacts on local processing in the striatum and potentially the fine-tuning of the drive of basal ganglia output, which serve as potential mechanisms of action of tTIS. Moreover, an additional characteristic of the axonal membrane, which is fundamental in tTIS, is the passive membrane filter property of neurons^57^. Currently, there is an open debate with alternative mechanistic hypotheses affirming the necessity of a rectification step before filtering to demodulate the electric field, thus allowing selective responses to the modulating envelope^58^.

So far, only a small number of published studies have applied tTIS in humans^59,60^. Nevertheless, they have targeted primary motor and parieto-occipital cortices, which are already reachable with conventional NIBS techniques. Studies targeting the motor cortex suggest that tTIS can obtain neural and behavioral effects, namely, tTIS can modulate functional rs-connectivity, induce faster reaction times and increase implicit motor learning^61,62^. Furthermore, Violante et al. suggested that theta-band tTIS applied to the hippocampus can modulate the activity and connectivity profile of the subcortical target structure and enhance episodic memory performance in young healthy subjects^63^. Importantly, electric field modeling and measurements in a human cadaver suggest that this effect is driven by focused stimulation of the hippocampus and minimizes tTIS exposure in the overlaying cortex.

Together with our results, this finding suggests that tTIS can focus on specific deep brain regions in human subjects without engaging overlaying cortices. These effects are induced by TI modulation and are independent of the HF content of the carrier signal. tTIS can modulate brain activity in the target region, the associated brain network and linked behavior. Importantly, striatal tTIS induces only minimal stimulation-associated sensations, has good blinding integrity and does not modulate subjects’ attention or fatigue levels (Supplementary Figs. 7 and 8), which is an important prerequisite in future controlled human neuroscience and clinical studies.

In addition to the current findings, several points should be addressed. First, even though the present intervention was applied in three cohorts, including both younger and older individuals, and across two experiments, interpretations of these findings need to consider the small sample size. Second, NIBS techniques show relevant intersubject variability in terms of response rates^64^. The degree of stimulation response variability during tTIS within and across subjects is currently unknown and should be addressed in future studies. It should be noted that we report rather small to medium effect sizes for the key neural and behavioral outcomes. When we benchmark tTIS to other low-intensity tES techniques, this is an order of magnitude that we would have expected for single-session interventions^65^. It may be speculated that the effect sizes are larger in conditions with aberrant brain network interactions (for example, in neurological disorders) or that they can be further augmented by increasing the dose of tTIS, for example, by multi-session application. Third, based on work suggesting mild effects of low-intensity kHz-frequency stimulation at the cortical level, we cannot completely rule out the possibility that a portion of the induced striatal neuromodulation effects was caused by the unmodulated HF signal^66^. However, the facts that the montage for the striatal target was optimized based on the modulated tTIS fields, that motor corticospinal excitability was not influenced by tTIS and that we could detect several significant contrasts between the tTIS and the HF control stimulation strongly suggest that the effects would more likely be explained by properties of the tTIS that are not present in HF control stimulation. Two are the possible differences: the exposure modulation amplitude or the offset in the mean frequency between the two stimulation protocols (tTIS carrier frequencies, *f*_1_ = 2.00 kHz, *f*_2_ = 2.10 kHz, *f*_mean_ = 2.05 kHz, unmodulated HF control frequency, *f*_1_ = *f*_2_ = *f*_mean_ = 2.00 kHz). For the last option to be true, the brain should show a frequency-dependent exposure distribution, meaning that exposure should sensibly depend on the carrier frequency. This scenario would require that the dielectric tissue properties vary importantly with frequency (dielectric dispersion). However, current tissue measurements indicate that no such variations occur in the low kHz range^67,68^. Hence, based on these observations, the behavioral and brain activity differences would more likely be explained by a decisive contribution of the time-modulated exposure magnitude. Furthermore, it was not within the scope of the study to investigate whether patterned theta-burst transcranial alternating current stimulation (tACS) protocols, which are modulated in the time domain similar to Kunz et al.^41^, can achieve comparable effects. However, the fact that the peak of the stimulation field of such protocols cannot be focused on deep brain regions without strongly stimulating the overlying cortex, along with their characteristic of only achieving mild and inhibitory effects^41^, makes this possibility very unlikely. Fourth, multiple groups have now highlighted the possibility of high-intensity, suprathreshold tTIS to generate conduction blocks^58,69^. However, the field strengths reached in the current experiments employing subthreshold, low-intensity tTIS are several orders of magnitude lower than the one employed in the aforementioned works, rendering such effects unlikely. Finally, in the present work, the optimized electrode montage was chosen on the basis of electric field distributions from simulations involving a detailed reference head model. However, there are important variabilities in anatomy and tissue properties, which can explain some of the observed response variability^70^. By using image-based and subject/patient-specific modeling to personalize electrode placement and stimulation parameters, it is likely that the selectivity and effectiveness of tTIS could be further optimized in the future^71,72^. Subject-specific, image-based information about brain anisotropy, for example, from diffusion tensor imaging (DTI), can also be used to consider the known^70,73^ impact of the relative orientation of the exposing field and the principal neural structures on stimulability^70^, in addition to the stimulability differences inherent to the different brain regions and neuron types.

To conclude, the present work reveals, for the first time in humans, the ability to noninvasively modulate neuronal activity in deep brain regions via theta-burst patterned striatal tTIS. The modulation led to increased activity not only in the targeted deep brain structure, namely the striatum, but also in the linked functional brain network. Furthermore, striatal theta-burst patterned tTIS induced significant behavioral improvements in a motor learning task, and this effect was especially pronounced in healthy older subjects.

In general, the proposed stimulation approach is a crucial step forward for the field of systems neuroscience, as it allows us to noninvasively characterize the effects of direct neuromodulation of deep brain activity. This approach thus suggests exciting opportunities for better understanding physiological and pathophysiological processes based on causal rather than associative evidence, for example, evidence derived using conventional neuroimaging techniques.

Overall, the proposed tTIS approach has high potential for noninvasively modulating and studying brain plasticity of deep brain structures in clinical contexts. This is of particular interest and importance as deep brain regions, such as the striatum, hippocampus and thalamus, play critical roles in various motor and cognitive functions and are key pathophysiological substrates in numerous neurological and psychiatric disorders, such as Alzheimer’s disease, Parkinson’s disease, stroke, addiction or anxiety disorders. To extend this proof-of-principle work, further investigations are required to evaluate underlying mechanisms, develop strategies for improving behavioral effects and establish pathways for personalized applications with the aim of translating this exciting, innovative approach to clinical settings.

## Methods

### Participants

Forty-five healthy participants were included in the two experiments. Based on prior studies investigating similar NIBS interventions combined with motor training^8,33,74^, we anticipated a large effect size of 0.8. Thus, we estimated the sample sizes based on a comparison of the pre/post stimulation differences between the conditions, with a level of significance of *P* < 0.05 (two-sided, matched pairs) and a power of 0.8. The estimation in GPower software^75^ suggested a sample size of 15 per group.

In experiment 1, 15 healthy young subjects (9 females, mean ± s.d. age 23.46 ± 3.66 years) were recruited. Fourteen out of 15 participants performed the full protocol, while one participant dropped out between sessions for personal reasons. Only the 14 full datasets were included in the analyses.

In experiment 2, 15 healthy older subjects (9 females, mean ± s.d. age 66.00 ± 4.61 years) and 15 healthy young subjects (8 females, mean ± s.d. age 26.67 ± 4.27 years) were recruited and completed the study.

In the TMS control experiment, eight healthy young subjects (four females, mean ± s.d. age 25.25 ± 3.01 years) were recruited.

All subjects self-reported being right-handed, and handedness was confirmed by the Edinburgh Handedness Inventory^76^ (Supplementary Table 5). The exclusion criteria are listed in Supplementary Table 6. The studies were conducted in accordance with the Declaration of Helsinki. All studies were approved by the Cantonal Ethics Committee Vaud, Switzerland (project number 2020-00127). All participants provided written informed consent. The participants received a monetary reimbursement of 20 CHF per hour for their time spent at the research center.

### Experimental protocol

Experiments 1 and 2, and the TMS control experiment, followed a double-blind, crossover design. The order of the experimental conditions followed a predefined pseudorandom sequence. A baseline visit was always performed after inclusion, including questionnaires summarized in Supplementary Table 5.

### Motor task

The motor task consisted of an established and widely used nine-digit SFTT^15,22^ implemented in the Presentation software (version 20.0, Neurobehavioral Systems). The subjects had to reproduce a sequence shown on a computer screen with their nondominant left hand by pressing a four-button box, with each finger corresponding to a specific number (from 2-index to 5-little finger; Fig. 5a). Oral and written instructions were provided, asking the participants to perform the task ‘as fast and as accurately as possible’ in the fixed period of 30 or 90 s provided for each block in Experiments 1 and 2, respectively, to prevent the participants from performing the task at the extremes of their individual speed–accuracy tradeoff. A dot was displayed below the number corresponding to the digit to be pressed, and the dot moved to the next digit as soon as a key was pressed, regardless of whether the correct key was chosen. No feedback about the correctness of the responses was provided. The block durations were chosen on the basis of the experimental conditions and the research question, for example, in the task-based fMRI experiment, we chose shorter block durations due to the high-pass filtering step in signal preprocessing^77^. Outside the scanner, a duration of 90 s was used, based on protocols used in our previous studies^22,29^. All sequences had an equivalent Kolmogorov complexity, which was determined on the basis of a well-established procedure^22^. The order of the applied sequences before and after the crossover was randomized and counterbalanced between subjects.

### Experiment 1

In experiment 1, four fMRI sessions were performed, with two rs and two task-based sessions with concomitant tTIS or HF control stimulation. For further details, please refer to the section below. Between sessions, we included a wash-out phase of at least 3 days (7.4 ± 4.2 days between resting state sessions and 10.3 ± 4.7 days between task-based fMRI sessions). During the rs-MRI sessions, functional images were acquired during three resting state sequences lasting 8 min each, namely before (pre), during and after (post) stimulation, while subjects fixated on a white cross on a black background. During the task-based fMRI sessions, the participants performed six 9-min 30-s training blocks with an approximately 1-min 30-s break between blocks (Fig. 5b). Each block included ten 30-s repetitions of the motor task (see ‘Motor task‘ under ‘Experimental protocol’) with the respective stimulation condition, alternated with 30 s of rest (fixation cross). All participants performed a short familiarization session outside the MRI environment and a 30-s baseline measurement inside the scanner before starting the training blocks. The baseline performance was verified to ensure that at least one entirely correct sequence was performed; otherwise, the baseline was repeated, and the new block was used for analysis instead of the first block. At the beginning of each of the four fMRI sessions, participants completed the Stanford Sleepiness Scale^78^ questionnaire to confirm that the subjects started the experiment at comparable levels of sleepiness across conditions (Supplementary Fig. 8). A VAS was employed to test subjects’ attention and fatigue before and after MRI acquisition. After each of the two study phases (that is, rs and task-based fMRI), we employed a standardized questionnaire adapted from Antal and colleagues^79^ to evaluate the sensations associated with tTIS and to quantify the efficiency of the blinding.

### Experiment 2

In experiment 2, two main training sessions were performed, with at least a 3-day wash-out period between sessions (9.5 ± 4.0 days between sessions for the younger cohort and 9.2 ± 3.9 days between sessions for the older cohort); for more details, see Fig. 5c. During each session, either tTIS or HF control stimulation was applied as participants performed seven 90-s blocks of the motor task (see ‘Motor task’ under ‘Experimental protocol’) alternated with 90-s breaks. In the central block, the order of requested button presses followed a predefined pseudorandom sequence to assess sequence-independent learning effects. A 90-s baseline block was acquired before training to assess initial individual performance, and three additional blocks were collected immediately (post), 90 min (FU1) and 24 h (FU2) after the stimulation. The baseline performance was investigated to ensure that at least one entirely correct sequence was performed; this effect was consistently achieved, and thus, additional repetitions were not needed. The subjects’ attention and fatigue levels were collected by having them complete VAS questionnaires (see above) before the baseline measurement, after the post measurement, and before and after each follow-up, while the Stanford Sleepiness Scale was completed before the baseline and follow-up assessments to confirm that the subjects began the experimental sessions at comparable levels of sleepiness across conditions. At the end of the second post measurement, participants were asked to complete the same sensation questionnaire as for experiment 1.

### TMS control experiment

To evaluate corticospinal excitability linked to the primary motor cortex (M1) before (Baseline), during (Stim), and after (Post) striatal tTIS or HF control stimulation, we performed a control experiment using TMS^80^ (see also Supplementary Fig. 2a). Single-pulse TMS was applied to the right M1. The experimental procedures are described in detail in our previous work^81^. In brief, we delivered monophasic pulses with a posterior-to-anterior direction in the underlying brain tissue with an orientation of ∼45° to the midsagittal line using a figure-of-eight coil (MC-B70 Butterfly Coil) connected to a MagPro X100 stimulator (MagVenture). The intensity was adjusted to 130% of the resting motor threshold at the baseline of each session and was kept constant throughout the experiment. The coil positioning was guided by a neuronavigation system (Localite). Motor evoked potentials were recorded from the abductor pollicis brevis muscle contralateral to stimulation. The surface electromyography sampling procedures are described in our prior work^81^. Twenty trials (intertrial jitter 7 s ± 30%) were recorded before, during and after 10 min of tTIS or HF control stimulation (randomized double-blind design). Eighty trials (intertrial jitter 6.90 s ± 30%), separated into four bins, were sampled during tTIS or HF control stimulation. All trials were visually inspected in Signal software (version 6.05, Cambridge Electronic Design). Trials were rejected on the basis of the following criteria (preprocessing): muscle preactivation exceeding ±25 μV from baseline <100 ms before the TMS pulse, technical artifacts or documented suboptimal coil placement during data acquisition. Data points containing fewer than six trials per stimulation condition after preprocessing were not considered for further analysis (9 out of 112 cases).

### tTIS

#### General concept

TI stimulation is a novel brain stimulation strategy that employs two or more independent stimulation channels delivering HF currents (oscillating at *f*_1_ and *f*_1_ + Δ*f*) within the kHz range, which are assumed to be inert in terms of inducing neuronal activity^6,82^. The two currents generate a modulated electric field, with the envelope oscillating at the low-frequency Δ*f* (target frequency) where the currents join or cross. The peak of the envelope amplitude can be steered toward target areas located deeper in the brain by tuning the electrode position and current ratio across stimulation channels; for further details, see ref. ^6^ and Fig. 5d. Based on these properties, TI stimulation can focally target deep structures without engaging overlying tissues. In the present work, we applied tTIS via surface electrodes, applying a low-intensity, subthreshold protocol respecting the currently accepted cutoffs and safety guidelines for low-intensity tES^79^.

### Stimulators

The tTIS currents were generated by two independent DS5 isolated bipolar constant current stimulators (Digitimer). The stimulation patterns were created using a custom-written MATLAB-based (MathWorks) graphical user interface and transmitted to the current sources using a standard digital-to-analog converter (DAQ USB-6216, National Instruments).

### Stimulation paradigms

We employed two stimulation conditions: active stimulation delivered in theta-bursts (tTIS) and a HF control. The control stimulation consisted of two oscillatory HF currents delivered at 2 kHz without any frequency shifts, which led to a flat envelope of HF exposure incapable of eliciting brain physiological responses, as suggested by previous work^6^.

tTIS was delivered in an intermittent pattern designed to mimic established theta-burst stimulation protocols, which were developed in hippocampal slice preparations^23^ and have been adopted in previous NIBS approaches^24,41^. These theta-burst stimulation protocols share two features of hippocampal physiology: (1) the presence of bursting patterns and (2) modulations at the theta frequency^48^. The chosen stimulation strategy thus differs importantly from that used in work on conventional (unpatterned) tTIS^60,61,83^, which, analogous to tACS protocols, is thought to mediate its effects by synchronizing neuronal oscillators to the stimulation frequency (neural entrainment)^52,60,62^. The aim of the theta-burst tTIS protocol employed here was to probe and support task-induced modulation of synaptic transmission efficiency. The stimulation pattern was derived from slice preparations^48^ and well-established TMS-based plasticity induction protocols^24^. Compared with TMS-based approaches, the intraburst frequency was increased from 50 to 100 Hz to more closely resemble protocols inducing LTP-like effects developed in slice preparations^48^. It should be noted that some features of the stimulation protocol used differed slightly from physiological theta-bursts. Examples of such deviations are the lack of modulation of the interpulse interval, the number or the amplitude of the pulses during the bursting events and the rigid theta frequency employed in the stimulation protocol^84,85^. Stimulation was applied using a novel pulsed stimulation approach that utilizes frequency modulation, changing one of the two carrier frequencies to switch between modulated and unmodulated exposure. This allowed us to achieve an arbitrary waveform pattern without the need to change the current amplitude. During tTIS, bursts of three pulses at 100 Hz were repeated every 200 ms (5 Hz, that is, the theta rhythm) for 2 s (train). To obtain this pattern, the first channel continuously delivered a current at a frequency *f*_1_ = 2 kHz, while the frequency of the second electrode pair was switched from *f*_1_ = 2 kHz to *f*_1_ + Δ*f* = 2.1 kHz every 200 ms for 30 ms during the 2-s trains to create pulses of 100 Hz. During the interburst and intertrain intervals (8 s), nonamplitude-modulated HF stimulation was applied (Fig. 5e).

The other stimulation parameters were set as follows: the current intensity per stimulation channel was set to 2 mA and kept constant across sessions and across subjects for the main experiments; pure stimulation duration 30 min (for experiment 1) and 10 min 30 s (for experiment 2), with breaks within each protocol; ramp-up/ramp-down period 5 s; electrode type: round, conductive rubber with conductive cream/paste; and electrode size 3 cm^2^. Only in the evaluation of perceived sensations, during the stimulation tests preceding the main experiments, the intensity per channel was increased in the following steps: 0.5 > 1 > 1.5 > 2 mA (see below and Supplementary Fig. 7).

For experiment 1, the stimulation was applied in the MRI environment by employing a standard radio frequency filter module and MRI-compatible cables (neuroConn). The technological, safety and noise tests and methodological factors are reported on the basis of the ContES Checklist^86^ in Supplementary Table 7.

### Modeling

Electromagnetic simulations were performed to identify the optimal electrode placement and current steering parameters. The simulations were performed using the MIDA head model^87^, which is a detailed anatomical head model featuring 117 distinguished tissues and regions that were derived according to multimodal image data of a healthy female volunteer. Importantly, in brain stimulation modeling, the model distinguishes different scalp layers, skull layers, gray and white matter, cerebrospinal fluid and the dura. Circular electrodes (*N* = 77, radius 7 mm) were placed on the skin according to the 10–10 system, and the electromagnetic exposure was determined using the ohmic-current-dominated electroquasistatic solver in Sim4Life version 5.0 (ZMT Zurich MedTech AG), which is suitable due to the dominance of ohmic currents over displacement currents and the long wavelength compared with the simulation domain. The dielectric properties were assigned according to the IT’IS Tissue Properties Database v4.0 (ref. ^67^). Rectilinear discretization was used, and grid convergence and solver convergence analyses were performed to ensure negligible numerical uncertainty, resulting in a grid that contained more than 54 M voxels. Dirichlet voltage boundary conditions were applied, followed by current normalization, and the electrode–head interface contact was treated as ideal. tTIS exposure was simulated for 1 mA current intensity and quantified according to the maximum modulation envelope magnitude formula proposed by Grossman et al. in ref. ^6^. The current amplitude used to optimize the position of the electrodes did not influence the optimization since the scaling of the current was equivalent to the scaling of the tTIS exposure distribution. Subsequently, a sweep over 960 permutations of the four electrode locations was performed, considering symmetric montages with parallel (sagittal, 729 configurations; coronal, 231 configurations) or crossing current paths, and the bilateral striatum (putamen, caudate and nucleus accumbens) exposure performance was quantified according to three metrics: (1) the target exposure strength, (2) focality ratio (the volume ratio of the target tissue above the threshold to the overall brain tissue above the threshold, which measures stimulation selectivity) and (3) activation ratio (the percentage of the target volume above the threshold, which measures target coverage). The threshold was defined as the 98% volumetric iso-percentile level of the tTIS. Two configurations were noted in the resulting pareto-optimal front: one that maximized focality and activation (pair 1: AF3 and AF4, pair 2: TP7 and TP8 montage; focality 30.3%, activation 28.2%, threshold 0.19 V m^−1^) and one that accepts a reduction of these two metrics by a quarter while increasing the target exposure strength by more than 50% (pair 1: F3 and F4, pair 2: TP7 and TP8; focality 23.9%, activation 22.1%, threshold 0.31 V m^−1^). As the latter montage predicted a larger stimulation intensity, this configuration of electrodes was selected to ensure that the target could be stimulated. Subsequently, the modeled electrodes were enlarged to match the size of the electrodes selected for the experiment (radius 9.8 mm), and a new tTIS exposure simulation was performed (Fig. 5f). Consistent with our previous findings for smaller electrodes, the new TI field predicted a high activation ratio (21.6%) and focality (22.4%) with a threshold equal to 0.29 V m^−1^.

### Comparing tTIS and tDCS

To find an optimal solution for striatum stimulation, we compared the predictions of the last tTIS simulation with the configuration of electrodes used to stimulate the motor system with transcranial direct current stimulation (tDCS)^88^. This tDCS montage assumed the application of two electrodes (4 × 4 cm^2^) located at the C3 (anodal) and Fp2 (cathodal) positions according to the 10-20 EEG system. To derive the electric field (E-field) generated with this setup, we used the same MIDA head model, Sim4Life solver, grid and tissue properties database as were used for the tTIS simulations. While the tTIS configuration was aimed at bilateral stimulation and the tDCS montage was used to affect the unilateral brain regions, we compared the resulting tTIS field and electric field in bilateral and unilateral structures. In particular, we calculated different parameters of the tTIS field for the bilateral striatum and a control region, namely the precentral gyri including the upper limb region, and the E-field generated with the tDCS montage for the left striatum and left precentral gyrus. As a result of this comparison, we concluded that the optimized tTIS configuration was more efficient for targeting the striatum (within striatum: mean ± s.d. 0.26 ± 0.04 V m^−1^, median 0.27 V m^−1^, 99th percentile 0.35 V m^−1^) than tDCS stimulation (within striatum: 0.17 ± 0.02 V m^−1^, median 0.17 V m^−1^, 99th percentile 0.25 V m^−1^). Additionally, according to the modeling predictions, the tTIS should be more focal due to a lower field generated in the precentral gyrus (mean ± s.d. 0.2 ± 0.04 V m^−1^, median 0.2 V m^−1^, 99th percentile 0.29 V m^−1^) than the tDCS montage (0.25 ± 0.04 V m^−1^, median 0.24 V m^−1^, 99th percentile 0.34 V m^−1^). The activation of the other brain regions, which excluded the target and control structures, was similar between the types of simulation (tTIS: mean ± s.d. 0.15 ± 0.06 V m^−1^; tDCS: 0.16 ± 0.14 V m^−1^). The location of the target and control regions was identified after coregistration of the MIDA brain with the BNA^27^.

### Electrode placement and review of tTIS-associated sensations

The stimulation electrode positions were defined on the basis of the above model and determined in the framework of the EEG 10–20 system^89^. The optimal positioning leading to the best stimulation of the target structure, that is, the bilateral striatum, included the following electrodes: F3, F4, TP7 and TP8. Their scalp locations were marked with a pen. After skin preparation (cleaned with alcohol), round conductive 3 cm^2^ rubber electrodes were placed by adding a conductive paste (Ten20, Weaver and Company, or Abralyt HiCl, Easycap GmbH) as an interface to the skin and held in position with tape. In experiment 1, the electrode cables were oriented toward the top of the head to allow good positioning inside the scanner, while in experiment 2, the electrode cables were oriented toward the bottom and fixed on the shoulders to prevent electrode displacement. The impedances were checked and optimized until they were less than 20 kΩ (ref. ^60^). Once good contact was obtained, the subjects underwent current intensity testing to be familiarized with the perceived sensations and to systematically document their reactions. The tTIS and HF control stimulation protocols were both applied for 20 s with increasing current amplitude per channel as follows: 0.5 mA, 1 mA, 1.5 mA and 2 mA. The participants were asked to report any kind of sensation, and if a sensation was felt, participants were asked to grade its intensity from 1 to 3 (light to strong) and to provide at least one adjective to describe the sensation (Supplementary Table 8). After this step, in experiment 1, the cables were replaced by MRI-compatible cables, and a bandage was added to apply pressure on the electrodes and keep them in place. In experiment 2, an EEG cap was used to hold the electrodes in place. The electrode impedances were measured before the current intensity testing, before the training with concomitant stimulation and after the intervention.

### Image acquisition

Structural and functional images were acquired using a 3-T MAGNETOM PRISMA scanner (Siemens). The 3D MPRAGE sequence was used to obtain T1-weighted images with the following parameters: repetition time (TR) 2.3 s, echo time (TE) 2.96 ms, flip angle 9°, number of slices 192, voxel size 1 × 1 × 1 mm, and field of view (FOV) 256 mm. Anatomical T2 images were collected with the following parameters: TR 3 s, TE 409 ms, flip angle 120°, number of slices 208, voxel size 0.8 × 0.8 × 0.8 mm and FOV 320 mm. Echo-planar imaging sequences were used to obtain functional images with the following parameters: TR 1.25 s, TE 32 ms, flip angle 58°, number of slices 75, voxel size 2 × 2 × 2 mm and FOV 112 mm.

### Image preprocessing

Functional imaging data were analyzed using Statistical Parametric Mapping 12 (SPM12; The Wellcome Department of Cognitive Neurology) implemented in MATLAB R2018a (MathWorks). All functional images underwent the same preprocessing, including the following steps: slice time correction, spatial realignment to the first image, normalization to the standard Montreal Neurological Institute (MNI) space and smoothing with a 6-mm full-width half-maximal Gaussian kernel. T1 anatomical images were coregistered to the mean functional image and then segmented to produce the forward deformation field used to normalize the functional images, allowing bias-corrected gray and white matter images to be obtained. Framewise displacement was calculated for each run to control head movement. The nonnormalized and normalized images were visually inspected to ensure good preprocessing quality. The signal-to-noise ratio was also computed to control for possible tTIS-related artifacts. The recon-all function of the FreeSurfer^90^ software was run, taking structural T1w and T2w images as inputs. For each individual subject, BNA parcellation was computed, and specific ROIs were then coregistered to the functional images and normalized to MNI space. A quality control of the preprocessing results was conducted. A threshold of 0.5 was chosen, and subjects showing more than 40% of voxels with framewise displacement higher than this threshold were discarded. In the current study cohort, no subject exceeded the limit value; thus, the whole dataset could be used. Furthermore, successful cleaning of the data was ensured by visually checking the preprocessing results. In particular, good registration between anatomical and functional images and normalization to standard space were checked.

### Signal-to-noise ratio

To verify the image quality and presence of possible artifacts due to concomitant stimulation, total signal-to-noise ratio maps were computed as the mean over the s.d. for each voxel time series. The average values of the spherical ROIs (10 mm radius) underneath the four electrodes used for tTIS and underneath the theoretical positions of four more distant electrodes were extracted (Supplementary Fig. 10). The locations of the spheres were derived by projecting the standard MNI coordinates on the scalp^91^ toward the center of the brain. The spheres were visually inspected to ensure that the whole volume was included in the brain. A linear mixed model was then used to investigate the effects of the stimulating electrodes versus those of the nonstimulating electrodes in the total signal-to-noise ratio maps.

### Data processing

#### rs-MRI

Independent component analysis-based artifact removal was performed on the preprocessed, smoothed images using the GIFT toolbox^92^. Twenty independent components were extracted and visually inspected to remove noise-related artifacts. Seed-based connectivity analyses were implemented at the single-subject level by extracting the average time series within the striatal mask defined in the BNA and including this time series as a regressor in a general linear model with six head motion parameters (three displacement motions and three rotation motions) and normalized time series in the white matter and corticospinal fluid. At the group level, a paired *t*-test was performed to compare striatal connectivity during the stimulation delivery period. Furthermore, a flexible factorial analysis was computed to investigate the presence of any effect on and among the prestimulation period, during stimulation and the poststimulation period. We included subject, stimulation and period (pre, during and post) as factors. Multiple comparison corrections were applied at the cluster level by controlling the FDR.

### Task-based fMRI

A general linear model was used to estimate the signal amplitude at the single-subject level. Six head motion parameters (three displacement motions and three rotation motions) and the normalized time series in the white matter and corticospinal fluid were included as regressors. Linear contrasts were computed to estimate activation during the motor task versus that during resting periods, and ROI-based analyses were conducted. An external radiologist manually drew striatal masks on each subject’s structural T1w image. After drawing the masks for the caudate and putamen, the anterior and posterior subparts were distinguished with respect to the location of the anterior commissure. Coregistration to the functional images and normalization to MNI space were then applied to obtain individual masks for each subject. The BOLD activity within the individual striatal masks was averaged and compared between different striatal subunits, namely the putamen versus the caudate, and within the putamen, namely the anterior putamen versus the posterior putamen.

Additionally, a flexible factorial design was used to compute group-level statistics, including subject, stimulation and time as factors. Multiple comparison corrections were applied at the cluster level by controlling the FDR, if not specified otherwise. ROI-to-ROI connectivity was computed via the CONN functional connectivity toolbox^93^.

### Motor task analysis

Behavioral data were analyzed with Python (version 3.8.3). In experiment 1, because of the relatively short duration of the motor task repetitions (30 s), motor learning was evaluated by extracting the number of correct key presses per repetition divided by the number of correct key presses during the baseline measurement^94^.

In experiment 2, motor learning was evaluated by extracting the number of correct sequences in each block divided by the correct number of sequences performed during the baseline measurement^22^. For the longer assessment blocks in experiment 2, sequence-based outcomes were chosen instead of key-press-based outcomes because these results more closely resemble the structure of natural skilled movements, which often require smaller elements of the movements to be performed in specific order and time sequences^95^.

In both cases, frame shifts in button pressing were considered, meaning that key presses that were performed in the correct order were considered correct even if the key presses did not match the dot indicating which digit to press next.

The baseline values were compared between the stimulation conditions and among sessions to assess comparable initial performance and to control for carry-over effects in each cohort; for more details, see Supplementary Fig. 5.

### Statistical analysis of the behavioral data

Statistical analyses were performed in the R software environment for statistical computing and graphics^96^. To analyze the behavioral motor learning data, we conducted linear mixed effects analyses employing the lmer function in the lme4 package^97^. As fixed effects, we added blocks and stimulation conditions to the model for experiment 1 and blocks, stimulation conditions and populations (younger and older) to the model for experiment 2. The subject factor was taken as a random intercept. Statistical significance was determined using the anova function with Satterthwaite’s approximations in the lmerTest package^98^. To mitigate the impact of isolated influential data points on the outcome of the final model, we employed tools in the influence.ME package to detect and remove influential points based on the following criterion: distance >4 × mean distance^99^. For the TMS control experiment, no correction for influential data points was carried out due to the smaller sample size. For specific post hoc comparisons, we conducted pairwise comparisons by computing the estimated marginal means using the emmeans package^100^. Effect size measures were obtained using the effectsize package^101^ and are expressed as p*η*^2^ values for the *F* tests and Cohen’s *d* values for pairwise comparison tests, corresponding to <0.01 (micro), 0.01 (small), 0.06 (medium), and 0.14 (large) effect sizes for p*η*^2^ and <0.2 (micro), 0.2–0.3 (small), 0.5 (medium), and 0.8 (large) effect sizes for *d* (ref. ^102^). The level of significance was set at *P* < 0.05. Residuals of the models were investigated to assess normality, skewness (between −2 and 2 (ref. ^103^)) and homoskedasticity. Finally, for the baseline and follow-up sessions, the normality of the data distribution was tested with the Shapiro‒Wilk test, and either paired *t*-tests or Wilcoxon rank sum tests were then used (two-sided). The functions were included in the stats package, which is part of the above referenced R software environment. A Bayes factor (BF_10_) was obtained via the JASP software^104^ (version 0.16.4) using the default parameters. The factor indicates that the data are 1/BF_10_ times more likely to occur under the null hypothesis (H0) than under the alternative hypothesis (H1). BF_10_ data suggest the following ranges^105^: 1–0.33 anecdotal, 0.33–0.1 moderate and 0.1–0.03 strong evidence in favor of H0.

### Reporting summary

Further information on research design is available in the Nature Portfolio Reporting Summary linked to this article.

## Online content

Any methods, additional references, Nature Portfolio reporting summaries, source data, extended data, supplementary information, acknowledgements, peer review information; details of author contributions and competing interests; and statements of data and code availability are available at 10.1038/s41593-023-01457-7.

### Supplementary information

Supplementary Information Supplementary Figs. 1–10 and Tables 1–8.

Reporting Summary

## Data Availability

All data necessary to generate the main results and figures are available in the Zenodo repository (10.5281/zenodo.8252501)^106^. The BNA was used and can be downloaded from http://atlas.brainnetome.org/.

## Code availability

The scripts necessary to generate the main results are available in the Zenodo repository (10.5281/zenodo.8252501)^106^.
