# Supplementary material for: Noninvasive theta-burst stimulation of the human striatum enhances striatal activity and motor skill learning
Source: Nat Neurosci. 2023 Oct 19;26(11):2005–16. doi: 10.1038/s41593-023-01457-7 (PMC10620076; doi:10.1038/s41593-023-01457-7)
Supplement: Supplementary file 1 — Supplementary Figs. 1–10 and Tables 1–8. [file 41593_2023_1457_MOESM1_ESM.pdf]

nature neuroscience

Article https://doi.org/10.1038/s41593-023-01457-7

Noninvasive theta-burst stimulation of the human striatum enhances striatal activity and motor skill learning

In the format provided by the authors and unedited

Set-level		Cluster-level				Peak-level					x	y	z	AAL3
p	c	$p_{FWE-corr}$	$q_{FDR-corr}$	k_E	p_{uncorr}	$p_{FWE-corr}$	$q_{FDR-corr}$	T	(Z_E)	p_{uncorr}				
**0.000**	**9**	**0.015**	**0.009**	**166**	**0.001**	**0.001**	**0.004**	**6.12**	**5.76**	**0**	**-64**	**-2**	**-4**	**Temporal_Sup_L**
						0.354	0.172	4.46	4.31	0	-58	12	-10	Temporal_Pole_Sup_L
						0.515	0.183	4.31	4.17	0	-62	-10	12	Rolandic_Oper_L
		**0.000**	**0.000**	**439**	**0.000**	**0.018**	**0.068**	**5.3**	**5.06**	**0**	**28**	**0**	**-22**	**Amygdala_R**
						0.124	0.151	4.79	4.61	0	30	6	-8	Putamen_R
						0.163	0.151	4.71	4.54	0	32	-2	-6	Putamen_R
		**0.012**	**0.008**	**174**	**0.001**	**0.03**	**0.073**	**5.18**	**4.95**	**0**	**10**	**-6**	**54**	**Supp_Motor_Area_R**
						0.587	0.209	4.25	4.12	0	-6	0	60	Supp_Motor_Area_L
						0.896	0.297	3.95	3.85	0	14	0	50	Supp_Motor_Area_R
		**0.001**	**0.001**	**289**	**0.000**	**0.164**	**0.151**	**4.71**	**4.54**	**0**	**-66**	**-22**	**36**	**Location not in atlas**
						0.187	0.152	4.67	4.5	0	-60	-22	30	Postcentral_L
						0.894	0.297	3.96	3.85	0	-60	-28	22	SupraMarginal_L
		**0.011**	**0.008**	**178**	**0.001**	**0.325**	**0.172**	**4.49**	**4.34**	**0**	**-56**	**4**	**34**	**Precentral_L**
						0.915	0.301	3.93	3.82	0	-46	0	40	Precentral_L
						0.999	0.558	3.56	3.48	0	-60	2	24	Precentral_L
		**0.009**	**0.008**	**184**	**0.000**	**0.354**	**0.172**	**4.46**	**4.31**	**0**	**10**	**-18**	**4**	**Thal_IL_R**
						0.469	0.172	4.35	4.21	0	14	-28	0	Thal_PuA_R
						0.977	0.376	3.78	3.69	0	18	-20	8	Thal_VPL_R
		**0.021**	**0.011**	**155**	**0.001**	**0.457**	**0.172**	**4.36**	**4.22**	**0**	**-42**	**-2**	**8**	**Insula_L**
						0.937	0.317	3.89	3.78	0	-50	0	4	Rolandic_Oper_L
						0.999	0.56	3.55	3.47	0	-36	6	-10	Insula_L
		**0.004**	**0.006**	**215**	**0.000**	**0.563**	**0.203**	**4.27**	**4.14**	**0**	**62**	**-10**	**12**	**Rolandic_Oper_R**
						0.76	0.262	4.1	3.98	0	54	-18	18	Rolandic_Oper_R
						0.845	0.279	4.02	3.9	0	62	-22	8	Temporal_Sup_R
		**0.005**	**0.006**	**207**	**0.000**	**0.641**	**0.223**	**4.21**	**4.08**	**0**	**-18**	**-50**	**-28**	**Location not in atlas**
						0.691	0.233	4.16	4.04	0	-30	-52	-26	Cerebellum_6_L
						0.957	0.334	3.84	3.74	0	-10	-50	-22	Cerebellum_4_5_L

Tab. S1: Summary of clusters showing higher BOLD activation for the tTIS vs. HF control contrast during the task-related fMRI experiment (Experiment 1). Significant clusters are shown for uncorrected p=0.001 at the voxel level, and FDR corrected p=0.05 at the cluster level.

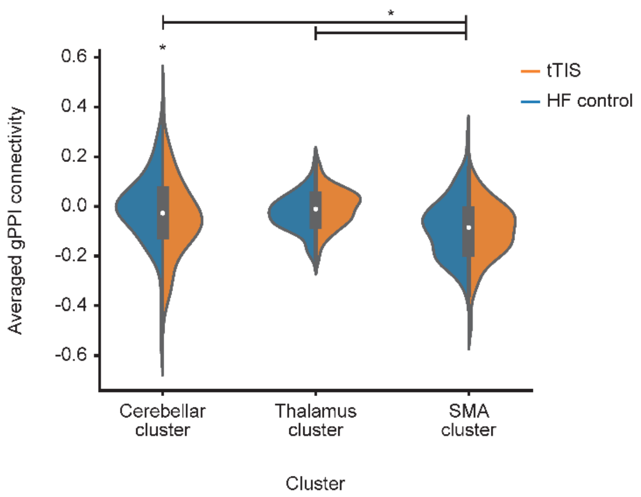

Fig. S1: Average region of interest (ROI) to ROI effective connectivity

Average ROI-to-ROI generalized psychophysiological interaction (gPPI) connectivity (Experiment 1, n=13, one influential point removed based on Cook's distance) from the right putamen to the clusters within the motor network (cerebellum, thalamus and SMA) showing a significant increase in activity during tTIS with respect to HF control stimulation was computed. A significant effect of cluster (one-sided ANOVA with Satterthwaite's approximations: $F(2, 450)=30.70$, $p=3.18e-13$, $p\eta^2=0.12$ *[medium]*) and a significant interaction stimulation x cluster (one-sided ANOVA with Satterthwaite's approximations: $F(2,450)=3.26$, $p=0.04$, $p\eta^2=0.01$ *[small]*) were observed. The white dot represents the median value, the grey boxplot represents the interquartile range and the line represents the 1.5 interquartile range. The cluster effect was driven by a lower connectivity observed from the putamen to the supplementary motor cortex with respect to the two other clusters. The interaction is explained by the presence of significantly lower connectivity induced by tTIS specifically between the right putamen and the cerebellar cluster. The decrease in putamen-cerebellar connectivity induced by tTIS could potentially explain a lowering of the inhibitory influence of the putamen on the cerbellum[1]. This would in turn lead to the increased BOLD activity observed in the cerebellum and the increased motor performance. To support this hypothesis, we repeated the behavioral modulation analysis considering all clusters showing higher activity during tTIS with respect to HF control stimulation. The results indicate that voxels within the cerebellum are significantly modulated by behavior as well. The observed correlation between behavior and cerebellar activity could hence be induced by the decrease in connectivity from the putamen. Taken together, these findings support the focal modulation of the striatum, which might lead to the difference in cerebellar activity associated with tTIS and to behavioral changes. The increased activity of the other motor-related areas might be more easily explained as a consequence of indirect striatal modulation and not as a result of higher behavioral performance in the tTIS condition. Otherwise, we would have expected a behavioral modulation, which was not present in this analysis.

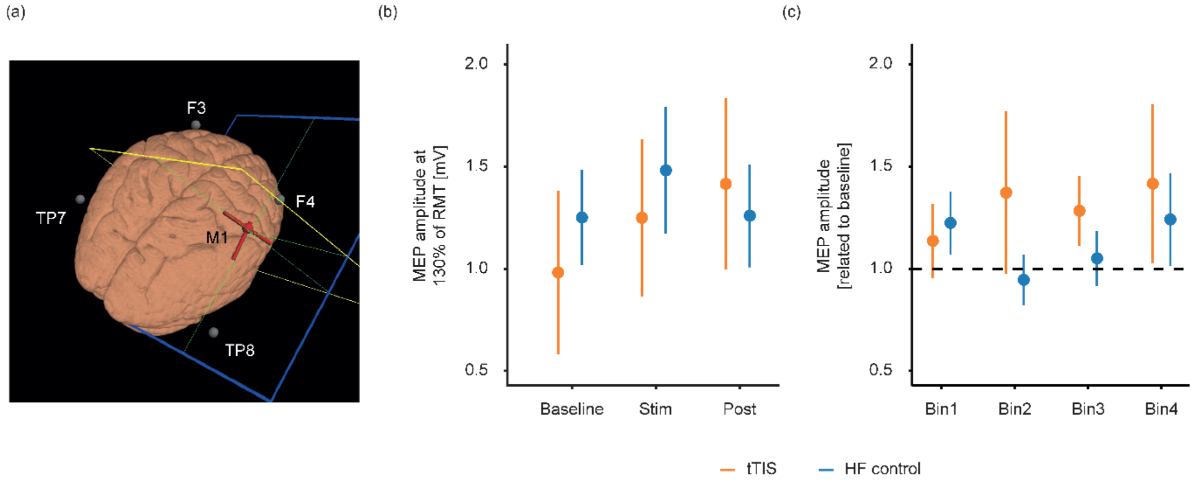

Fig. S2. Control experiment - no effects on corticospinal excitability linked to primary motor cortex (M1)

N=8 young, healthy subjects were recruited. **(a)** MEPs induced by TMS of the right M1 were sampled from the abductor pollicis brevis muscle (APB) before (Baseline), during (Stim), or after (Post) tTIS or HF control stimulation to quantify a surrogate marker of corticospinal excitability. TMS intensity was adjusted to 130% of the resting motor threshold (RMT) before the start of the baseline evaluation and kept constant throughout the experiment. **(b)** Averaged MEP amplitude per block sampled before (Baseline), during (Stim), or after (Post) 10 min of tTIS or HF control stimulation. No effects on corticospinal excitability were detected (one-sided ANOVA with Satterthwaite's approximations: stimulation x timing: *$F(2,34.03)=1.19$, $p=0.32$ $p\eta^2=0.07$ [medium], $BF_{10}=0.36$, [anecdotal evidence for H0]*). **(c)** Evaluation of the evolution of the MEP amplitude during tTIS or HF control stimulation. Data were divided into 4 bins (20 trials per bin). No effects on corticospinal excitability were detected (one-sided ANOVA with Satterthwaite's approximations: stimulation x bin: *$F(3,40.71)=0.46$, $p=0.71$, $p\eta^2=0.03$ [small], $BF_{10}=0.05$, [strong evidence for H0]*). The dots indicate the measure of center (mean value across the stimulation condition) and the error bars are standard errors (SEs). The results indicate that neither striatal tTIS nor HF control stimulation modulate corticospinal excitability linked to M1. This suggests that striatal tTIS does not directly coactivate the overlying M1.

Measure	NumDF	DenDF	F	p	pη^2	BF_{10}
Contrast before, during, or after tTIS or HF control stimulation						
Stimulation	1	34.03	0.89	0.35	0.03	0.69
Timing	2	34.03	1.58	0.22	0.09	0.64
Stimulation x timing	2	34.03	1.19	0.32	0.07	0.36
Contrast during tTIS or HF control stimulation						
Stimulation	1	41.97	0.10	0.76	0.002	0.61
Bin	3	40.70	0.64	0.60	0.04	0.21
Stimulation x bin	3	40.71	0.46	0.71	0.03	0.05

Tab. S2. Summary of statistical analyses for the TMS control experiment
In the first step, an ANOVA with Satterthwaite's approximations was used to evaluate the significance of the employed linear mixed model effects (see the methods section). In this control experiment, no influential point analysis was performed due to the small sample size. The BF_{10} column depicts the Bayes factor.

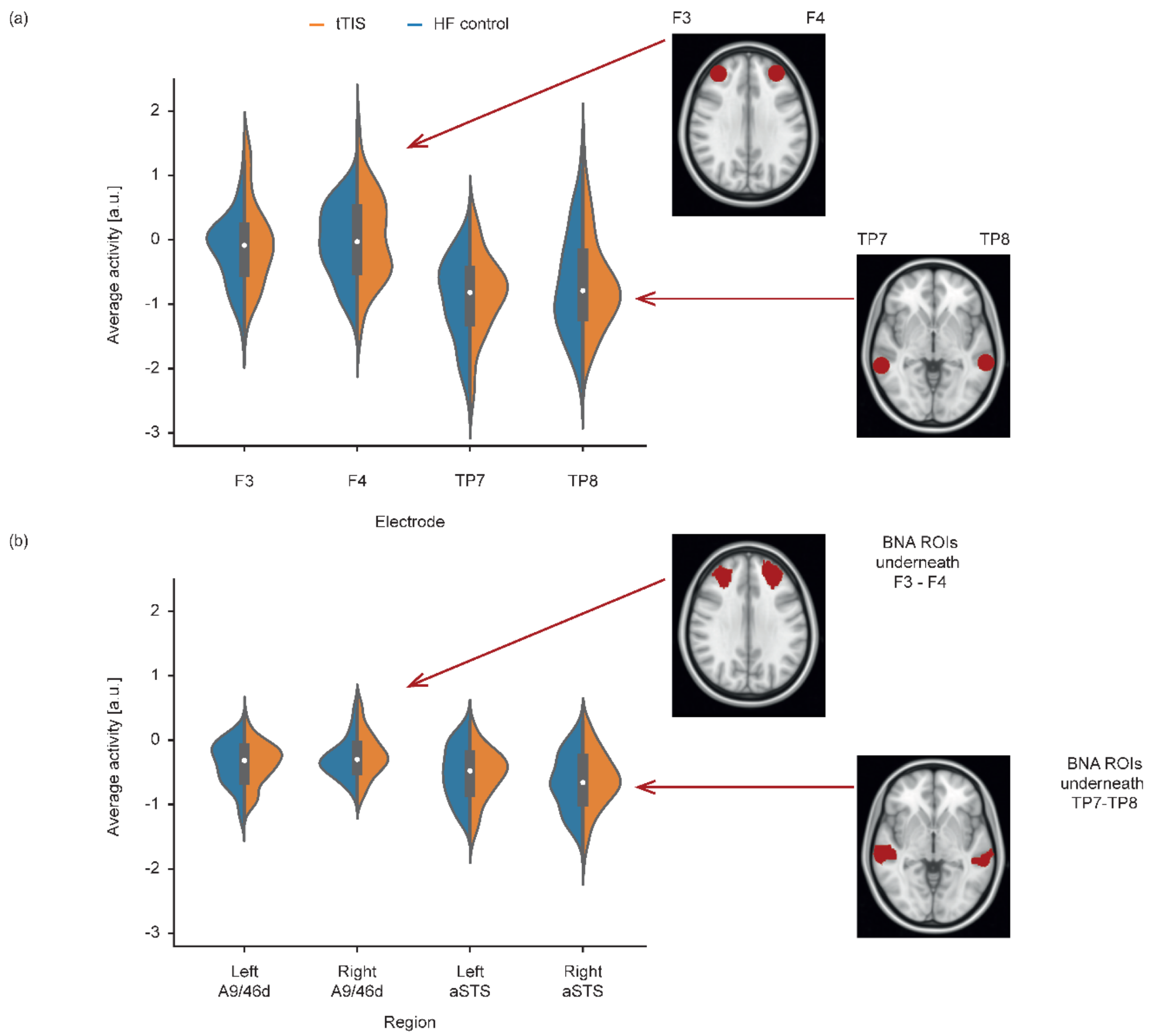

Fig. S3: BOLD activity in control regions beneath the electrodes
BOLD activity during the task-based fMRI experiment (Experiment 1, n=14) was extracted and averaged across blocks from **(a)** spheres (radius=10 mm) placed below the stimulation electrodes (EEG 10-10 positions: F3, F4, TP7, and TP8) or **(b)** corresponding regions based on the Brainnetome Atlas (BNA)[2] (the left and right dorsal area A9/46d for the F3/F4 or the left and right anterior superior temporal sulcus (aSTS) for the TP7/TP8 positions). Please note that the values are negative as the activity during the task is referenced to activity during rest periods, in which the subjects kept their eyes open and fixated on a cross, following a standard block design procedure. Statistical difference between the two stimulation conditions was tested via a one-sided ANOVA with Satterthwaite's approximations. Sphere model: $F(1,651)=2.04$, $p=0.15$, $p\eta^2=0.003$ *[micro]*; BNA model $F(1,651)=0.38$, $p=0.54$, $p\eta^2=0.0006$ *[micro]*. In all violin plots: the white dot represents the median value, the grey boxplot represents the interquartile range and the line represents the 1.5 interquartile range.

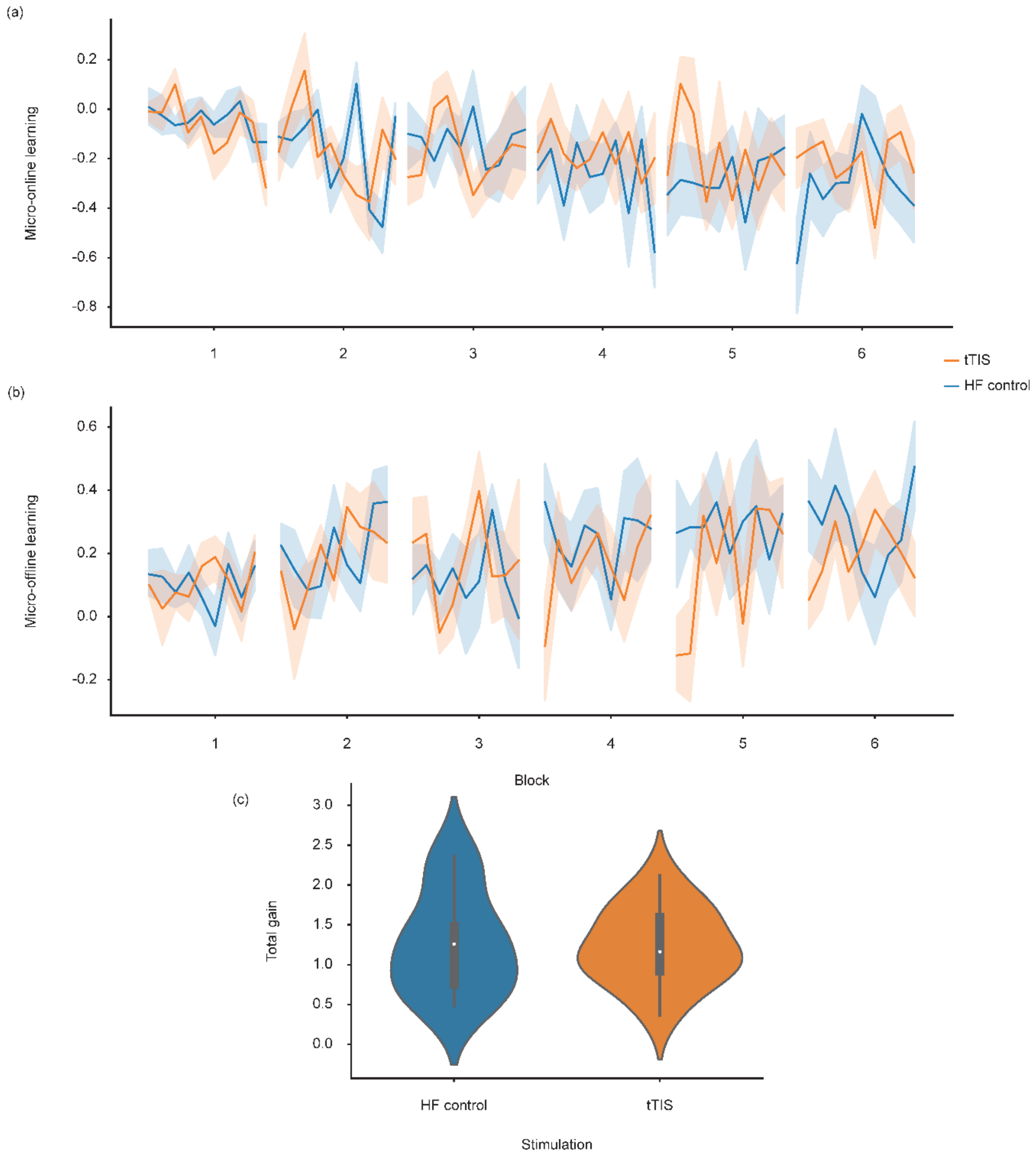

Fig. S4: Micro-online learning, micro-offline learning and total gain in Experiment 1
Micro-online and micro-offline learning measures were computed following prior studies analyzing this outcome for the sequential finger tapping task[3,4]. Nevertheless, because of the longer duration of our blocks (30 instead of 10 seconds), the longer length of the sequence (9 versus 5 digits) and our primary outcome measure (correct key presses versus tapping speed), micro-offline and micro-online learning were extracted based on the number of correct key presses within the first and last 10 seconds of each repetition. We computed the number of correct key presses in the first and last 10 seconds of each 30-second repetition of the task of each MRI block. We then defined micro-online learning as the difference in the number of correct key presses between the last and first 10 seconds within the same repetition, while micro-offline learning was computed by subtracting the number of correct key presses in the last 10 seconds of the previous repetition from the first 10 seconds of the current repetition. **(a)** Micro-online learning computed as the difference between the number of correct key presses, baseline corrected, in the last and first 10 seconds within the same repetition. No significant difference was observed between stimulation protocols (one-sided ANOVA with Satterthwaite's approximations: $F(1,1547)=1.98$, $p=0.16$, $p\eta^2=0.001$ *[micro]*). The lines indicate the measure of center (mean value across the

stimulation condition) and the shadow areas represent standard errors (SEs). **(b)** Micro-offline learning computed by subtracting the number of correct key presses, baseline corrected, during the last 10 seconds of the previous block minus the ones during the first 10 seconds of the current block. No significant difference was observed between stimulation protocols (one-sided ANOVA with Satterthwaite's approximations: $F(1,1521)=2.36$, $p=0.12$, $p\eta^2=0.002$ *[micro]*). The lines indicate the measure of center (mean value across the stimulation condition) and the shadow areas represent standard errors (SEs). **(c)** Total gain (Experiment 1, n=14) computed as the difference between the number of correct key presses, baseline corrected, during the last repetition of the task versus that during the first repetition. No significant difference was observed between stimulation protocols (two-sided paired t-test: $t(13)=0.23$, $p=0.82$, $d=0.06$). The white dot represents the median value, the grey boxplot represents the interquartile range and the line represents the 1.5 interquartile range.

Set-level		Cluster-level				Peak-level					x	y	z	AAL 3
p	c	$p_{FWE\text{-}corr}$	$q_{FDR\text{-}corr}$	k_E	p_{uncorr}	$p_{FWE\text{-}corr}$	$q_{FDR\text{-}corr}$	T	(Z_E)	p_{uncorr}				
**0.000**		**0.000**	**0.004**	**197**	**0.000**	**0.133**	**0.076**	**8.35**	**4.83**	**0**	**28**	**4**	**-4**	**Putamen_R**
						0.910	0.366	6.06	4.11	0	26	16	2	Putamen_R
						0.985	0.530	5.58	3.92	0	28	4	10	Putamen_R

Tab. S3: Summary of clusters showing stronger modulation of BOLD activity by behavioral performance within the right striatum. Significant clusters are shown for uncorrected p=0.001 at the voxel level, and FDR corrected p=0.05 at the cluster level.

Older cohort						
Contrast	Estimate	SE	Df	t.ratio	p.value	d
0 - 1	-0.15	0.11	351	-1.33	0.84	-0.34
0 - 2	-0.31	0.11	351	-2.76	0.09	-0.71
0 - 3	-0.39	0.11	351	-3.38	0.01	-0.87
0 - 4	-0.42	0.11	351	-3.68	0.005	-0.95
0 - 5	-0.57	0.11	351	-5.03	1.6e-05	-1.30
0 - 6	-0.67	0.11	351	-5.91	1.7e-07	-1.53
1 - 2	-0.16	0.11	351	-1.43	0.78	-0.37
1 - 3	-0.23	0.11	351	-2.06	0.38	-0.53
1 - 4	-0.27	0.11	351	-2.35	0.22	-0.61
1 - 5	-0.42	0.11	351	-3.71	0.005	-0.96
1 - 6	-0.52	0.11	351	-4.59	1.3e-04	-1.18
2 - 3	-0.07	0.11	351	-0.62	1.00	-0.16
2 - 4	-0.10	0.11	351	-0.92	0.97	-0.24
2 - 5	-0.26	0.11	351	-2.27	0.26	-0.59
2 - 6	-0.36	0.11	351	-3.15	0.03	-0.81
3 - 4	-0.03	0.11	351	-0.30	1.00	-0.08
3 - 5	-0.19	0.11	351	-1.65	0.65	-0.43
3 - 6	-0.29	0.11	351	-2.53	0.15	-0.65
4 - 5	-0.15	0.11	351	-1.35	0.83	-0.35
4 - 6	-0.25	0.11	351	-2.23	0.28	-0.58
5 - 6	-0.10	0.11	351	-0.88	0.98	-0.23
**Young cohort**						
0 - 1	-0.16	0.12	351	-1.34	0.83	-0.36
0 - 2	-0.47	0.12	351	-4.02	0.001	-1.07
0 - 3	-0.62	0.12	351	-5.26	5.2e-06	-1.41
0 - 4	-0.72	0.12	351	-6.14	4.8e-08	-1.64
0 - 5	-0.97	0.12	351	-8.23	0.0e+00	-2.20
0 - 6	-1.11	0.12	351	-9.44	0.0e+00	-2.52
1 - 2	-0.32	0.12	351	-2.68	0.11	-0.72
1 - 3	-0.46	0.12	351	-3.92	0.002	-1.05
1 - 4	-0.57	0.12	351	-4.80	4.8e-05	-1.28
1 - 5	-0.81	0.12	351	-6.90	5.3e-10	-1.84
1 - 6	-0.95	0.12	351	-8.10	0.0e+00	-2.17
2 - 3	-0.15	0.12	351	-1.24	0.88	-0.33
2 - 4	-0.25	0.12	351	-2.12	0.34	-0.57
2 - 5	-0.50	0.12	351	-4.21	6.3e-04	-1.13
2 - 6	-0.64	0.12	351	-5.42	2.3e-06	-1.45
3 - 4	-0.10	0.12	351	-0.88	0.98	-0.23
3 - 5	-0.35	0.12	351	-2.97	0.05	-0.79
3 - 6	-0.49	0.12	351	-4.18	7.2e-04	-1.12
4 - 5	-0.25	0.12	351	-2.10	0.36	-0.56
4 - 6	-0.39	0.12	351	-3.30	0.02	-0.88
5 - 6	-0.14	0.12	351	-1.21	0.89	-0.32

Tab. S4: Experiment 2 – supplementary results: block x population interaction. Pairwise comparisons by computing the estimated marginal means using Tukey adjustment.

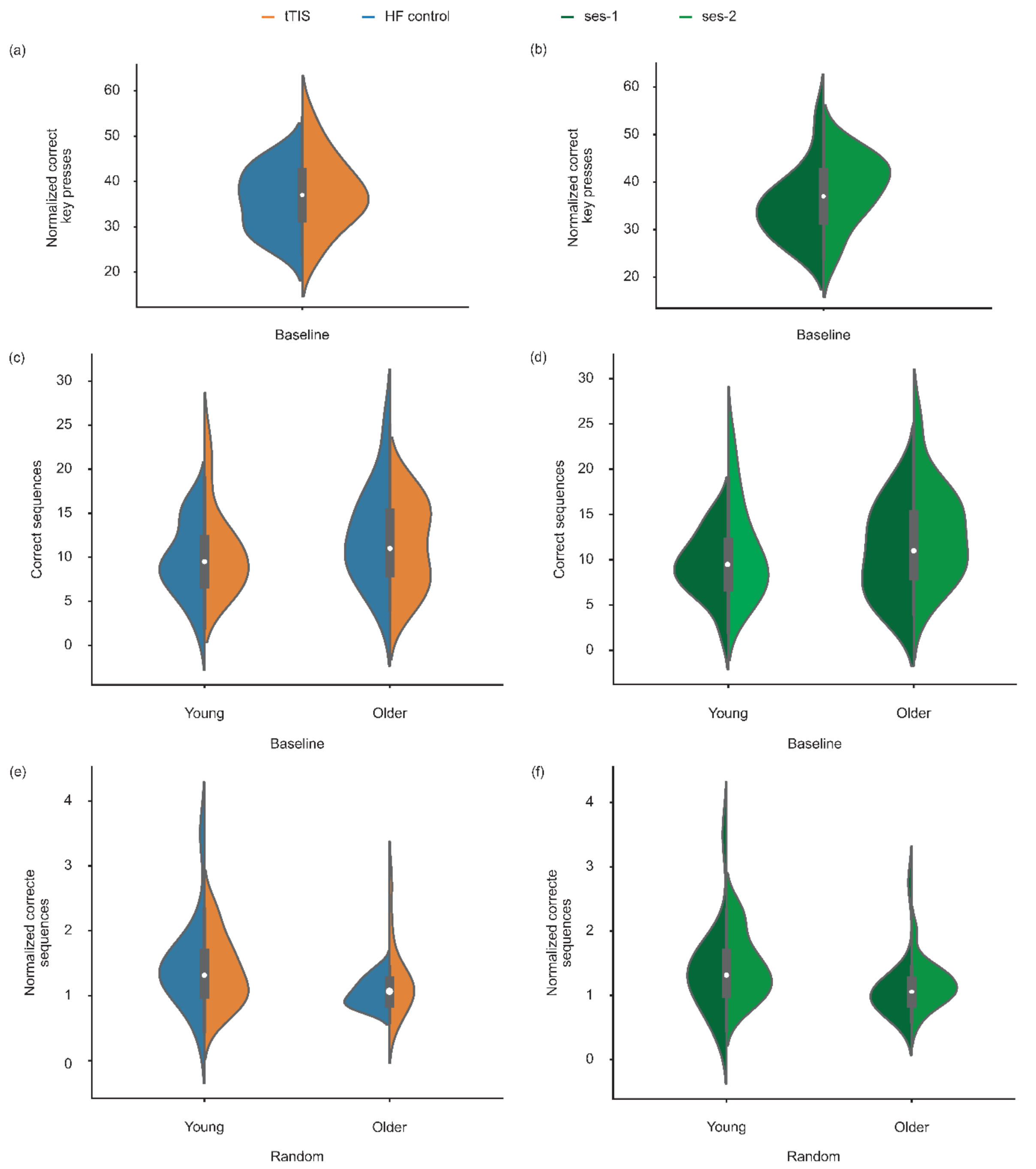

Fig. S5: Motor performance at baseline or during pseudorandom sequences

(a) Comparison of baseline performance between tTIS and HF control stimulation in Experiment 1 (n=14). No significant difference was found (two-sided paired t-test *t(13)=0.29, p=0.78, d=0.08; Bayesian paired t test,* BF_{10}*=0.28* [*moderate evidence in favor of the null hypothesis (*H_0*)*]). **(b)** Comparison of baseline performance between the first and second training sessions in Experiment 1 (n=14). A significant difference was found, with higher performance during the second session (two-sided paired t-test *t(13)=-3.35, p=0.005, d=-0.77; Bayesian paired t test,* BF_{10}*=9.77 [moderate evidence in favor of the alternative hypothesis (*H_1*)]*). **(c)** Comparison of baseline performance between tTIS and HF control stimulation in Experiment 2; data for the young cohort (n=15) are shown on the left and data for the older cohort (n=15) are shown on the right. No significant difference was found (two-sided paired t-test, young: *t(14)=0.55, p=0.59, d=0.22; Bayesian paired t test,* BF_{10}*=0.37 [anecdotal evidence for* H_0*];* two-sided paired t-test, older: *t(14)=-0.55, p=0.59, d=-0.21; Bayesian paired t test,* BF_{10}*=0.37 [anecdotal evidence for* H_0*]*). **(d)** Comparison of baseline performance between

the first and second training sessions in Experiment 2; data for the young cohort (n=15) are shown on the left and data for the older cohort (n=15) are shown on the right. No significant difference was found in the young cohort (two-sided paired t-test, *t(14)=-1.44, p=0.17, d=-0.34; Bayesian paired t test,* BF_{10}*=0.62 [anecdotal evidence for* H_0*]*), while a marginally significant increase in performance was observed in the older cohort during the second session (two-sided paired t-test, *t(14)=-2.10, p=0.05, d=-0.45; Bayesian paired t test,* BF_{10}*=1.44 [anecdotal evidence for* H_1*]*). **(e)** Comparison of performance during a pseudorandom motor sequence between tTIS and HF control stimulation in Experiment 2; data for the young cohort (n=15) are shown on the left and data for the older cohort (n=15) are shown on the right. No significant difference was found (two-sided paired Wilcoxon test, young: *V=53, p=0.72, d=-0.19; Bayesian paired t test,* BF_{10}*=0.29 [moderate evidence for* H_0*];* two-sided paired Wilcoxon test, older: *V=82, p=0.23, d=0.44; Bayesian paired t test,* BF_{10}*=0.56 [anecdotal evidence for* H_0*]*). **(f)** Comparison of performance during a pseudorandom motor sequence between the first and the second training session in Experiment 2; data for the young cohort (n=15) are shown on the left and data for the older cohort (n=15) are shown on the right. No significant difference was found (two-sided paired Wilcoxon test, young: *V=50, p=0.60, d=-0.10; Bayesian paired t test,* BF_{10}*=0.27 [moderate evidence for* H_0*];* two-sided paired Wilcoxon test, older: *V=32, p=0.21, d=-0.17; Bayesian paired t test,* BF_{10}*=0.29 [moderate evidence for* H_0*]*).

In all violin plots, the white dot represents the median value, the gray boxplot represents the interquartile range, and the line represents the 1.5 interquartile range.

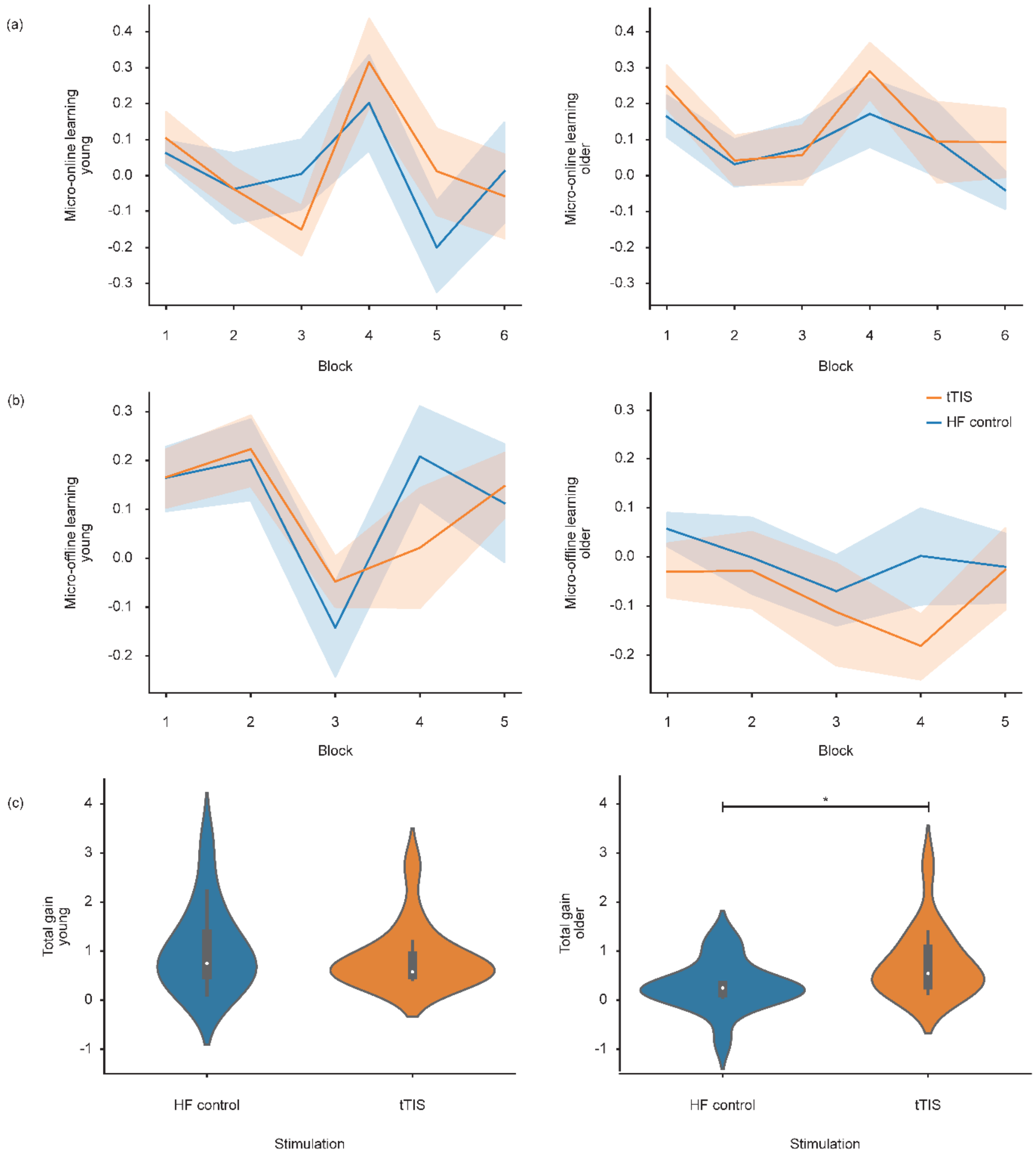

Fig. S6: Micro-offline learning, micro-online learning and total gain in Experiment 2

Micro-online and micro-offline effects in Experiment 2 were computed by extracting the number of correct key presses in the first and last 10 seconds of each training block. (**a**) Micro-online learning, in the young cohort on the left and in the older cohort on the right, computed as the difference between the number of correct key presses, baseline corrected, in the last and first 10 seconds within the same block. No significant effect of stimulation was found (one-sided ANOVA with Satterthwaite's approximations: $F(1,308)=1.20, p=0.27, p\eta^2=0.004$ *[micro]*). The lines indicate the measure of center (mean value across the stimulation condition) and the shadow areas represent standard errors (SEs). **(b)** Micro-offline learning, in the young cohort on the left and in the older cohort on the right, computed by subtracting the number of correct key presses, baseline corrected, during the last 10 seconds of the previous block from those during the first 10 seconds of the current block. No significant effect of stimulation was found (one-sided ANOVA with Satterthwaite's approximations: $F(1,243)=1.26, p=0.26, p\eta^2=0.005$ *[micro]*). The lines indicate the measure of center (mean value across the stimulation condition) and the shadow areas represent standard errors (SEs). In line with previous work[5], we found a significant difference in offline learning between the young and older cohorts (two-

sided pairwise comparisons via estimated marginal means: *t(27)=-2.25, p=0.033, d=-0.5 , Tukey adjustment*), whereby the older cohort showed a reduced offline learning. **(c)** Total gain, in the young cohort (n=15) on the left and in the older cohort (n=15) on the right, computed as the difference between the number of correct key presses, baseline corrected, during the last block of the task versus that during the first block. A significantly higher total gain was observed in the older cohort when tTIS was applied with respect to that in response to HF control stimulation (two-sided paired Wilcoxon test: *V=96, p=0.041, d=0.76*). No significant effect of stimulation was found in the young cohort (two-sided paired Wilcoxon test: *V=40, p=0.28, d=-0.39*). The white dot represents the median value, the grey boxplot represents the interquartile range and the line represents the 1.5 interquartile range.

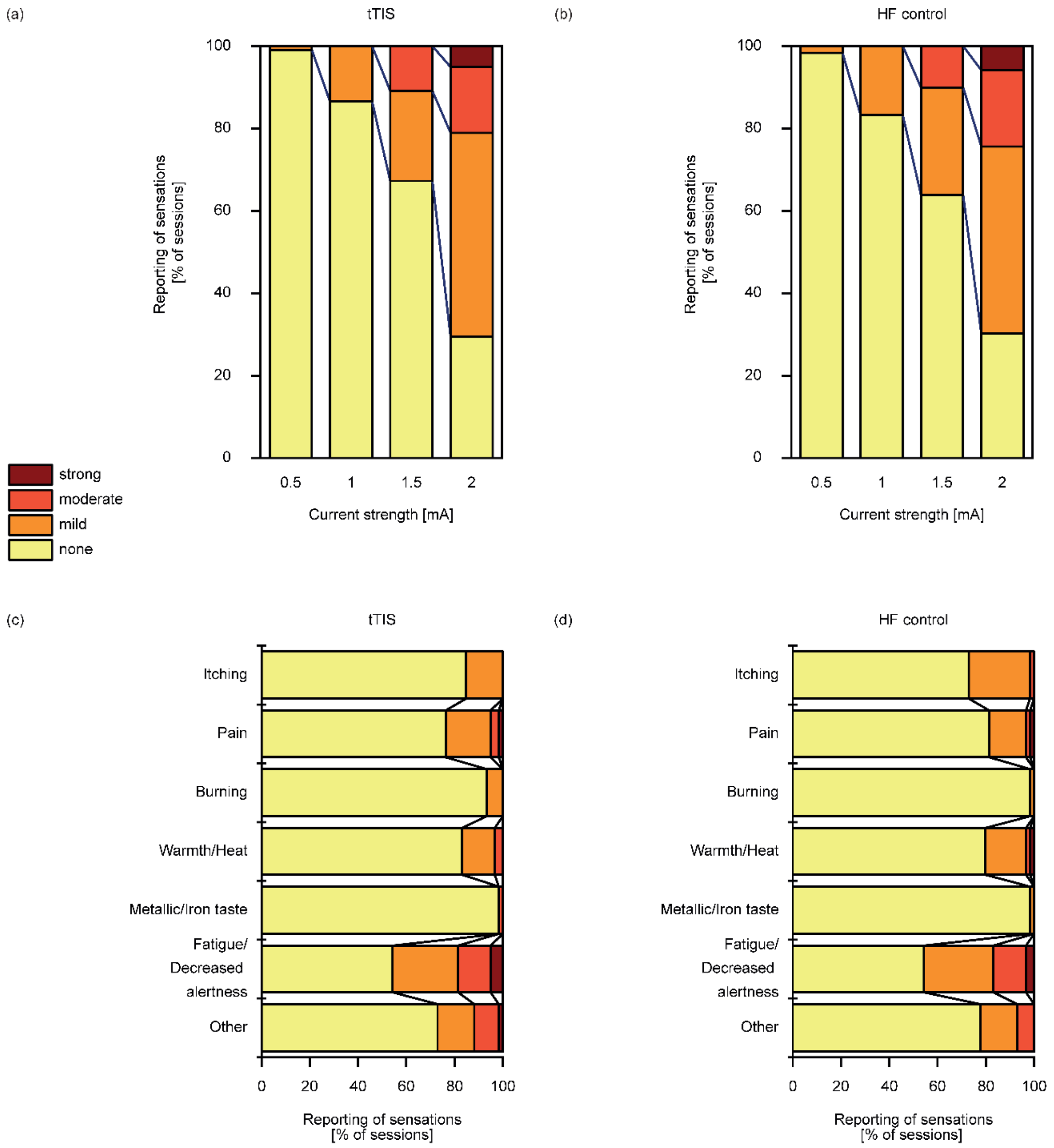

Fig. S7: tTIS-associated sensations

(a) and **(b)** The frequency of reported sensations separated by their strength (none, mild, moderate, and strong) are depicted for the tested current intensity levels (0.5, 1.0, 1.5, 2 mA per stimulation channel) during the stimulation-associated sensation tests preceding a total of N=119 experimental sessions. No differences in perceived stimulation-associated sensations could be detected when comparing the two tested conditions, tTIS and HF control, as indicated by a nonsignificant stimulation condition x current strength interaction (one-sided ANOVA with Satterthwaite's approximations: $F(3,837.95)=0.06$, $p=0.98$, $p\eta^2=0.0002$ *[micro]* $BF_{10}=0.001$, *[decisive evidence for H0]*). **(c) and (d)** Systematic evaluation of the perceived sensations during the interventional sessions (N=118) revealed no stimulation condition-associated differences for the commonly tested tES protocol-associated sensations[6] (stimulation condition x sensation category (one-sided ANOVA with Satterthwaite's approximations: $F(6,727.26)=0.73$, $p=0.63$, $p\eta^2=0.006$ *[micro]*, $BF_{10}=0.0009$ *[decisive evidence for* H_0*]*). In an additional step, we systematically asked the subjects to provide their best guess on the applied stimulation condition. The analysis of the tTIS sessions suggested that in both Experiment 1 and Experiment 2, the frequencies of correct and incorrect responses were not significantly different

from each other (exact binomial test for (i) rs-fMRI: $p=0.55$, (ii) task-based fMRI: $p=0.75$, (iii) young subjects in the behavioral experiment: $p=1.00$, and (iv) older subjects in the behavioral experiment: $p=1.00$). In conclusion, the analyses showed excellent blinding integrity of the novel tTIS protocol with respect to the HF control condition.

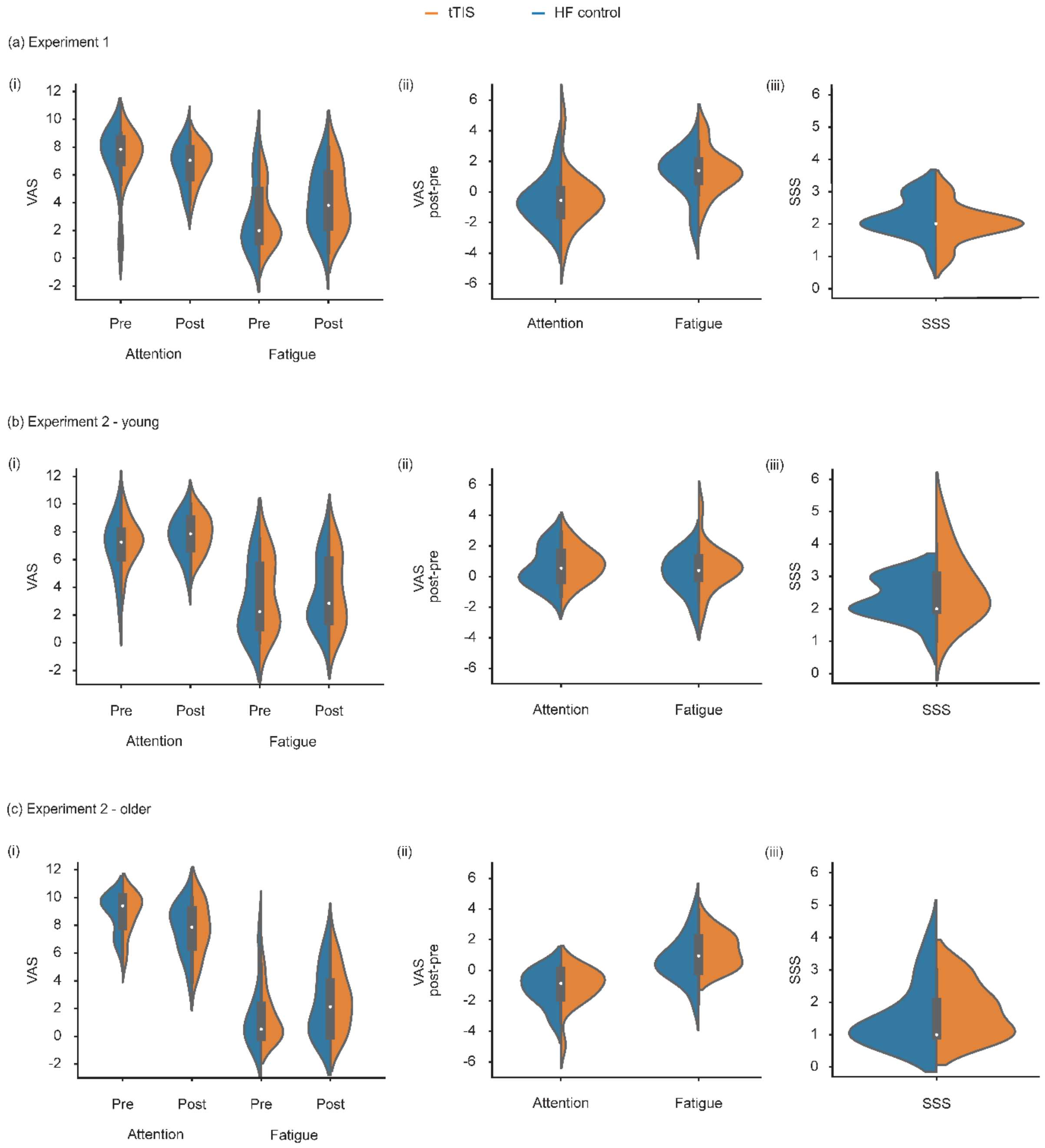

Fig. S8: Questionnaire responses about attention, fatigue and sleepiness

(a) Subjects' level of attention and fatigue during Experiment 1 (n=14), quantified with a visual analog scale (VAS), ranging from 0 to 10. (i) Comparison of attention and fatigue levels between tTIS and HF control stimulation sessions, pre- and posttraining. No significant difference was found (two-sided paired Wilcoxon test, attention pre: *V=54, p=0.95*; two-sided paired t- test, attention post: *t(13)=-0.06, p=0.95*; two-sided paired Wilcoxon test, fatigue pre: *V=50.5, p=0.93*; two-sided paired t- test, fatigue post: *t(13)=-0.24, p=0.82*). (ii) Comparison of changes in attention and fatigue levels pre- and posttraining between tTIS and HF control stimulation sessions. No significant difference was found (two-sided paired Wilcoxon test, attention: *V=45, p=0.66*; two-sided paired t- test, fatigue: *t(13)=-0.77, p=0.46*). (iii) Comparison of sleepiness, quantified with the Stanford Sleepiness Scale (SSS)[7], between tTIS and HF control stimulation sessions before training. No significant difference was found (two-sided paired Wilcoxon test, *V=24, p=0.41*). **(b)** Attention and fatigue level in Experiment 2 in the young population (n=15); the same assessment method as in Experiment 1 was used. (i) Comparison of

attention and fatigue levels between tTIS and HF control stimulation sessions, pre- and posttraining. No significant difference was found (two-sided paired t- test, attention pre: *t(14)=0.54, p=0.60*; attention post: two-sided paired t- test, *t(14)=0.36, p=0.73*; two-sided paired Wilcoxon test, fatigue pre: *V=68.5, p=0.65*; two-sided paired Wilcoxon test, fatigue post: V*=63, p=0.89*). (ii) Comparison of changes in attention and fatigue levels pre- and posttraining between tTIS and HF control stimulation sessions. No significant difference was found (two-sided paired t- test, attention: *t(14)=-0.54, p=0.60*; two-sided paired t- test, fatigue: *t(14)=-1.02, p=0.32*). (iii) Comparison of sleepiness between tTIS and HF control stimulation sessions before training. No significant difference was found (two-sided paired Wilcoxon test, V=16, p=0.24). **(c)** Attention and fatigue level in Experiment 2 in the older population (n=15). (i) Comparison of attention and fatigue levels between tTIS and HF control stimulation sessions, pre- and posttraining. No significant difference was found (two-sided paired Wilcoxon test, attention pre: *V=53, p=1.00*; two-sided paired t- test, attention post: *t(14)=0.06, p=0.95*; two-sided paired Wilcoxon test, fatigue pre: *V=65.5, p=0.78*; two-sided paired Wilcoxon test, fatigue post: *V=56, p=0.85*). (ii) Comparison of changes in attention and fatigue levels pre- and posttraining between tTIS and HF control stimulation sessions. No significant difference was found (two-sided paired Wilcoxon test, attention: *V=35, p=0.48*; two-sided paired t- test, fatigue: *t(14)=-1.55, p=0.14*). (iii) Comparison of sleepiness between tTIS and HF control stimulation sessions before training. No significant difference was found (two-sided paired Wilcoxon test, V=22.5, p=0.64). In all violin plots, the white dot represents the median value, the gray boxplot represents the interquartile range, and the line represents the 1.5 interquartile range.

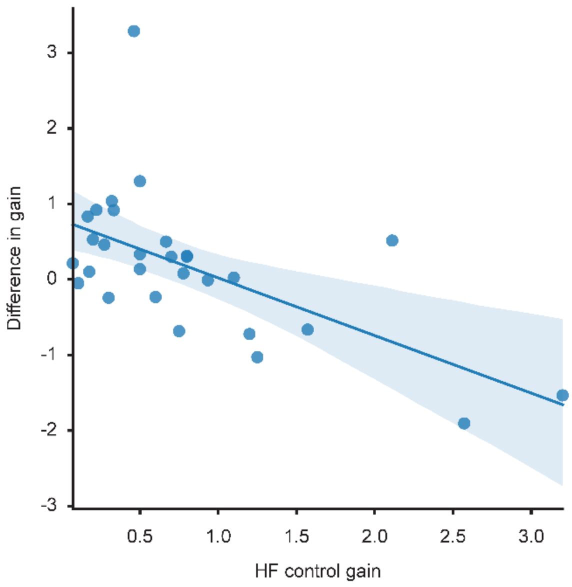

Fig. S9: Correlation between HF control gain and tTIS improvement in gain

To investigate the hypothesis that stimulation effects depend on natural motor performances, we investigated the relationship between the natural gain (gain during the control stimulation) and the improvements in gain between tTIS and the control stimulation. A linear model merging both the young and older cohorts from Experiment 2 was computed. A significant effect of the natural gain was observed on the stimulation effect (one-sided ANOVA: *F(1)=15.24, p=0.0006,* $p\eta^2$*=0.36 [large]*). Shadow area represents 95% confidence interval.

Experiment	Age	Sex	EHI	PSQI	FAB	MOCA
#1	23.46 ± 3.66	8/15 females	87.68 ± 16.59	4.50 ±1.79	n/a	n/a
#2 (older subjects)	66.00 ± 4.61	9/15 females	89.00 ± 14.47	3.47 ± 1.81	16.20 ± 2.11	27.67 ± 1.76
# 2 (young subjects)	26.67 ± 4.27	9/15 females	79.26 ± 23.82	5.67 ± 1.95	n/a	n/a

Tab. S5: Overview of participants' characteristics
EHI: Edinburgh Handedness Inventory[8]; PSQI: Pittsburg Sleep Quality Index[9]; FAB: Frontal Assessment Battery[10] (only assessed for the older cohort); MOCA: Montreal Cognitive Assessment[11] (only assessed for the older cohort).

Exclusion criteria
• Unable to consent<br>• Severe neuropsychiatric (e.g., major depression or severe dementia) or unstable systemic diseases (e.g., severe progressive and unstable cancer or life-threatening infectious diseases)<br>• Severe sensory or cognitive impairment or musculoskeletal dysfunctions prohibiting instruction comprehension or experimental task performance<br>• Inability to follow or noncompliance with the procedures of the study<br>• Contraindications for noninvasive brain stimulation (NIBS) or MRI:<br>o Electronic or ferromagnetic medical implants/device; non-MRI compatible metal implant<br>o History of seizures<br>o Medications that significantly interact with NIBS are benzodiazepines, tricyclic antidepressants and antipsychotics<br>• Regular use of narcotic drugs<br>• Left-handedness<br>• Pregnancy<br>• Request to not be informed in case of incidental findings<br>• Concomitant participation in another trial involving neuronal plasticity probing

Tab. S6: Study participant selection criteria

ContES Checklist	
**Technological factors**	
Manufacturer of the Stimulator	DS5 Isolated Bipolar Constant Current Stimulator *(Digitimer Ltd, Welwyn Garden City, UK).*
MR Conditional Electrodes	Round, 3 cm^2 conductive rubber electrodes.
Electrode Positioning	F3>F4<br>TP7>TP8<br><br>A bandage is wrapped around the head to apply pressure and keep the electrodes in place.<br><br>Electrodes are oriented in order to have vertical cables entering parallel to the MRI coil.<br><br>Head was fixed with pillows to avoid movements.
MR Conditional Skin-Electrode Interface	Ten20 conductive paste *(Weaver and Company, Aurora, CO, USA).*<br><br>One or two drops of saline were added when impedance was too high.
Amount of Contact Medium (Paste/Gel/Electrolyte)	Approximately 1 mm of paste was manually placed on the electrodes.
Electrode Placement Visualization	Pictures<br>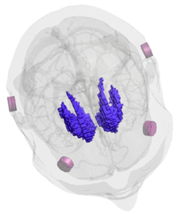
RF Filter	NeuroConn DC-STIMULATOR MR RF filter module with MRI-compatible cables and electrodes *(neuroConn GmbH, Ilmenau, Germany).*
Wire Routing Pattern	10 m ethernet cables between the inner and outer box pass through a conduit along the wall of the MRI room until reaching the back of the MRI scanner. Cables are then fixed with straps on the ground and on the wall of the MRI machine in

	order to avoid loops until reaching the interior of the coil.<br><br>Cables between the head and the inner boxes were also fixed with straps and they were oriented in order to minimize the magnetic field influence as much as possible, as indicated by the red arrows in the image below.<br><br>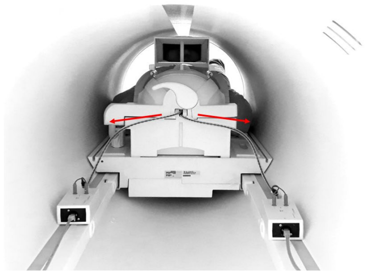
tES-fMRI Machine Synchronization/Communication	Stimulation was triggered by the stimulus delivery PC via parallel port to the BNC cable. The parallel port of the stimulus delivery PC was connected to the DAQ controlling the stimulators. The stimulus delivery PC, in turn, also received the scanner trigger from the scanner via USB port.
**Safety and noise tests**	
MR Conditionality Specifics for tES Setting	Please refer to the Section *"Methods: Image acquisition"*.
tES-fMRI Setting Test – Safety Testing	Impedances were checked before and after the stimulation.<br><br>No temperature tests were performed during the experiment.<br><br>Intensity titration was performed prior to entering the MRI scanner, testing increasing currents (0.5, 1, 1.5 and 2 mA) and asking the subject to report any type of sensation.<br><br>A sensation questionnaire was also completed at the end of the experiment.
tES-fMRI Setting Test – Subjective Intolerance Reporting	No intolerances were reported by any subject.
tES-fMRI Setting Test – Noise/Artifact	Signal-to-noise ratio (SNR) analyses were performed on the fMRI data; please refer to the Supplementary Fig. S10 below.

Impedance Testing	Impedance was checked right after electrode positioning outside the scanner, and before and after the stimulation inside the scanner.<br><br>One or two drops of saline solution were added if impedance was higher than 20 kΩ.
**Methodological factors**	
Concurrent tES-fMRI Timing	For timings, please refer to Fig. 1b in the main text of the manuscript.
Imaging Session Timing	All sequences were performed with tTIS electrodes placed on the subjects' heads.
tES Experience Report	Please refer to the Supplementary Fig. S7 above.

Tab. S7: ContES Checklist
Reporting on technological, safety and noise tests, and methodological factors based on the ContES Checklist[12].

Experiment	HF control	tTIS
#1	• Tingling<br>• Warm/Heat<br>• Burning<br>• Shiver<br>• Vibration<br>• Pain<br>• Waves<br>• Itching<br>• Scratching<br>• Pressure<br>• Massaging<br>• Tickling<br>• Tiny needles	• Tingling<br>• Warm<br>• Shiver<br>• Vibration<br>• Pain<br>• Waves<br>• Itching<br>• Scratching<br>• Pressure<br>• Massaging<br>• Tickling<br>• Tiny needles<br>• Slight touch<br>• Stinging
#2	*Young subjects:*<br>• Tingling<br>• Pressure<br>• Slight touch<br>• Warm<br>• Pinching<br>• Tickling<br>• Ants<br>• Massaging<br>• Contracting<br>• Pins and needles<br>• Drilling<br>• Vibration<br>• Pulsating<br><br>*Older subjects:*<br>• Warm<br>• Oscillation<br>• Tingling<br>• Pain<br>• Tickling<br>• Pressure	*Young subjects:*<br>• Tingling<br>• Pressure<br>• Pinching<br>• Tickling<br>• Ants/like a small insect moving<br>• Contracting<br>• Drilling<br>• Vibration<br>• Shiver<br>• Goose bumps<br><br>*Older subjects:*<br>• Warm<br>• Oscillation<br>• Tingling<br>• Tickling<br>• Pressure

Tab. S8: tTIS-associated sensations: subjective quality based on subjects' reports

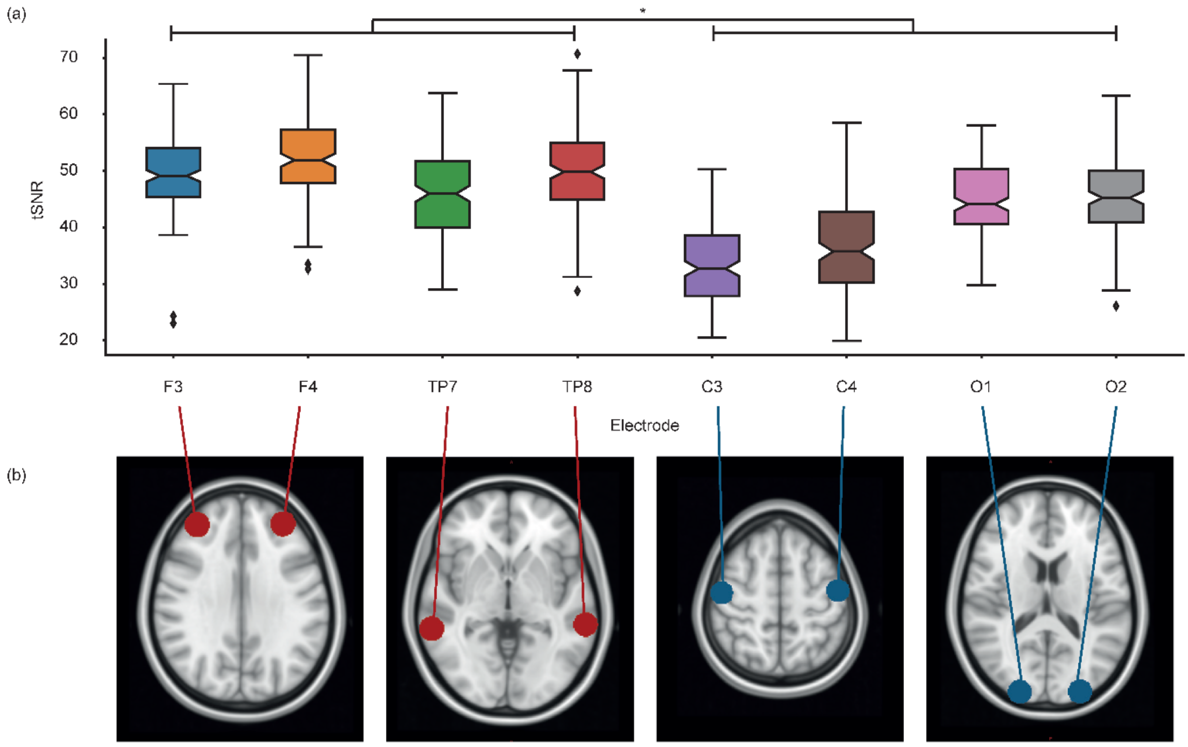

Fig. S10: Signal-to-noise ratio analysis

Total signal-to-noise ratio (tSNR) maps were created to investigate possible stimulation-induced artifacts. tSNR values were obtained as the ratio between the mean and the standard deviation of the time series during each fMRI block for each subject. An average value was then extracted from spheres with radii of 10 mm underneath each of the four stimulation electrodes and underneath the other four control electrode positions (not used in the study). **(a)** N=14, 2 sessions with 6 scans each. tSNR average values are plotted for each of the electrode-associated spheres. Stimulation electrodes showed higher tSNRs with respect to the control electrodes (one-sided ANOVA with Satterthwaite's approximations: $F(1,1323)=564.68$, $p=3.08e-104$, $p\eta^2=0.30$ *[large]*), indicating that no additional noise was introduced by the stimulation. This result indicates that the stimulation did not introduce additional noise in the MR images, since this would have led to lower SNR values. **(b)** Images showing the position of the chosen spheres underneath the used electrodes (in red) and underneath the control positions (in blue). In all box plots, the crossbar represents the median value, colored areas represent the interquartile range, and the whiskers represent the 1.5 interquartile range. Diamonds show individual points outside the 1.5 interquartile range.

References

1. Liebrand, M. *et al.* Beneficial effects of cerebellar tDCS on motor learning are associated with altered putamen-cerebellar connectivity: A simultaneous tDCS-fMRI study. *Neuroimage* **223**, 117363 (2020).
2. Fan, L. *et al.* The Human Brainnetome Atlas: A New Brain Atlas Based on Connectional Architecture. *Cereb Cortex* **26**, 3508–3526 (2016).
3. Bönstrup, M. *et al.* A Rapid Form of Offline Consolidation in Skill Learning. *Curr Biol* **29**, 1346-1351.e4 (2019).
4. Bönstrup, M., Iturrate, I., Hebart, M. N., Censor, N. & Cohen, L. G. Mechanisms of offline motor learning at a microscale of seconds in large-scale crowdsourced data. *NPJ Sci Learn* **5**, 7 (2020).
5. Maceira-Elvira, P. *et al.* Dissecting motor skill acquisition: Spatial coordinates take precedence. *Sci. Adv.* **8**, eabo3505 (2022).
6. Antal, A. *et al.* Low intensity transcranial electric stimulation: Safety, ethical, legal regulatory and application guidelines. *Clin Neurophysiol* **128**, 1774–1809 (2017).
7. Hoddes, E., Zarcone, V., Smythe, H., Phillips, R. & Dement, W. C. Quantification of Sleepiness: A New Approach. *Psychophysiology* **10**, 431–436 (1973).
8. Oldfield, R. C. The assessment and analysis of handedness: the Edinburgh inventory. *Neuropsychologia* **9**, 97–113 (1971).
9. Buysse, D. J., Reynolds, C. F., Monk, T. H., Berman, S. R. & Kupfer, D. J. The Pittsburgh Sleep Quality Index: a new instrument for psychiatric practice and research. *Psychiatry Res* **28**, 193–213 (1989).
10. Dubois, B., Slachevsky, A., Litvan, I. & Pillon, B. The FAB: a Frontal Assessment Battery at bedside. *Neurology* **55**, 1621–1626 (2000).
11. Nasreddine, Z. S. *et al.* The Montreal Cognitive Assessment, MoCA: a brief screening tool for mild cognitive impairment. *J Am Geriatr Soc* **53**, 695–699 (2005).
12. Ekhtiari, H. *et al.* A checklist for assessing the methodological quality of concurrent tES-fMRI studies (ContES checklist): a consensus study and statement. *Nat Protoc* **17**, 596–617 (2022).
