# Supplementary material for: Noninvasive theta-burst stimulation of the human striatum enhances striatal activity and motor skill learning
Source: Nat Neurosci. 2023 Oct 19;26(11):2005–16. doi: 10.1038/s41593-023-01457-7 (PMC10620076; doi:10.1038/s41593-023-01457-7)
Supplement: Supplementary file 2 — Reporting Summary [file 41593_2023_1457_MOESM2_ESM.pdf]

nature portfolio

Corresponding author(s): Friedhelm C. Hummel

Last updated by author(s): Sep 2, 2023

Reporting Summary

Nature Portfolio wishes to improve the reproducibility of the work that we publish. This form provides structure for consistency and transparency in reporting. For further information on Nature Portfolio policies, see our Editorial Policies and the Editorial Policy Checklist.

Statistics

For all statistical analyses, confirm that the following items are present in the figure legend, table legend, main text, or Methods section.

n/a	Confirmed	
☐	☒	The exact sample size (n) for each experimental group/condition, given as a discrete number and unit of measurement
☐	☒	A statement on whether measurements were taken from distinct samples or whether the same sample was measured repeatedly
☐	☒	The statistical test(s) used AND whether they are one- or two-sided<br>*Only common tests should be described solely by name; describe more complex techniques in the Methods section.*
☐	☒	A description of all covariates tested
☐	☒	A description of any assumptions or corrections, such as tests of normality and adjustment for multiple comparisons
☐	☒	A full description of the statistical parameters including central tendency (e.g. means) or other basic estimates (e.g. regression coefficient) AND variation (e.g. standard deviation) or associated estimates of uncertainty (e.g. confidence intervals)
☐	☒	For null hypothesis testing, the test statistic (e.g. F, t, r) with confidence intervals, effect sizes, degrees of freedom and P value noted<br>*Give P values as exact values whenever suitable.*
☐	☒	For Bayesian analysis, information on the choice of priors and Markov chain Monte Carlo settings
☒	☐	For hierarchical and complex designs, identification of the appropriate level for tests and full reporting of outcomes
☐	☒	Estimates of effect sizes (e.g. Cohen's d, Pearson's r), indicating how they were calculated

Our web collection on statistics for biologists contains articles on many of the points above.

Software and code

Policy information about availability of computer code

Data collection	Behavioral data: Presentation software (version 20.0, Neurobehavioral Systems, Berkeley, CA, USA)<br>MRI data: 3T MAGNETOM PRISMA scanner (Siemens, Erlangen, Germany)<br>TMS data: Signal software (version 6.05, Cambridge Electronic Design Ltd., Milton, England)
Data analysis	Behavioural data: Python (version 3.8.3), R software environment for statistical computing and graphics (version 4.1.3, https://www.r-project.org/), JASP software (version 0.16.4, https://jasp-stats.org).<br>MRI data: Statistical Parametric Mapping 12 (SPM12; https://www.fil.ion.ucl.ac.uk/spm/) implemented in MATLAB R2018a (Mathworks, Sherborn, MA, USA), Freesurfer (coded in Bash, 4.4.20(1)-release, CONN functional connectivity toolbox (20.b), and Python (version 3.8.3), https://surfer.nmr.mgh.harvard.edu/)<br>TMS data: Signal software (version 6.05, Cambridge Electronic Design Ltd., Milton, England), R software environment for statistical computing and graphics (version 4.2.1, https://www.r-project.org/), JASP software (version 0.16.4, https://jasp-stats.org).<br><br>Code availability:<br>The scripts necessary to generate the main results are available in the Zenodo repository (10.5281/zenodo.8252501).

For manuscripts utilizing custom algorithms or software that are central to the research but not yet described in published literature, software must be made available to editors and reviewers. We strongly encourage code deposition in a community repository (e.g. GitHub). See the Nature Portfolio guidelines for submitting code & software for further information.

Data

Policy information about availability of data

All manuscripts must include a data availability statement. This statement should provide the following information, where applicable:

- Accession codes, unique identifiers, or web links for publicly available datasets
- A description of any restrictions on data availability
- For clinical datasets or third party data, please ensure that the statement adheres to our policy

Data availability:
All data necessary to generate the main results and figures are available in the Zenodo repository (10.5281/zenodo.8252501). The Brainnetome atlas was used and can be downloaded from: http://atlas.brainnetome.org/.

Research involving human participants, their data, or biological material

Policy information about studies with human participants or human data. See also policy information about sex, gender (identity/presentation), and sexual orientation and race, ethnicity and racism.

Reporting on sex and gender	In Experiment 1:<br>- 15 healthy young subjects, 9 females, 6 males<br>In Experiment 2:<br>- 15 healthy older subjects, 9 females, 6 males<br>- 15 healthy young subjects, 8 females and 7 males<br>In TMS control experiment:<br>- 8 healthy young subjects, 4 females, 4 males.<br>Sex was determined based on self-reporting.<br>We did not consider the factor sex in the analyses.
Reporting on race, ethnicity, or other socially relevant groupings	No grouping was applied in the current study.
Population characteristics	In Experiment 1, 15 healthy young subjects (9 females, mean±SD age 23.46±3.66 years) were recruited. Fourteen out of 15 participants performed the full protocol, while one participant dropped out between sessions for personal reasons. Only the 14 full datasets were included in the analyses.<br>In Experiment 2, 15 healthy older subjects (9 females, average age 66.00±4.61 years) and 15 healthy young subjects (8 females, average age 26.67±4.27 years) were recruited and completed the study.<br>In the TMS control experiment, 8 healthy young subjects (4 females, average age 25.25±3.01 years) were recruited and completed the study.
Recruitment	For recruitment of participants, we distributed flyers at Campus Biotech in Geneva and other public places.<br>The procedure was supported via an information sheet. Flyers and the information sheet were approved by the ethics committee.<br>Selection bias: Healthy young subjects were recruited to a significant part within the university community through verbal or written advertisements. This entails that a disproportionately high number of subjects with a high level of education were recruited. To minimize the impact of this selection bias, the study employed a randomized cross-over design and we distributed the advertisement also at other public places.
Ethics oversight	Cantonal Ethics Committee Vaud, Switzerland (project number 2020-00127).

Note that full information on the approval of the study protocol must also be provided in the manuscript.

Field-specific reporting

Please select the one below that is the best fit for your research. If you are not sure, read the appropriate sections before making your selection.

☒ Life sciences ☐ Behavioural & social sciences ☐ Ecological, evolutionary & environmental sciences

For a reference copy of the document with all sections, see nature.com/documents/nr-reporting-summary-flat.pdf

Life sciences study design

All studies must disclose on these points even when the disclosure is negative.

Sample size	Based on prior studies investigating similar non-invasive brain stimulation interventions combined with motor training (Hashemirad et al. 2016, Brain and Cognition; Kang et al. 2016, JNNP; Zimerman et al. 2013, Annals of Neurology), we anticipated a large effect size of 0.8. Thus, we estimated the sample sizes based on a comparison of the pre/post stimulation differences between the conditions, with a level of significance of $p < 0.05$ (two-sided, matched pairs) and a power of 0.8. The estimation in GPower software (version 3.1.9.7, http://

www.gpower.hhu.de/) suggested a sample size of 15 per group.

Data exclusions

One participant dropped out between sessions for personal reasons. Imaging data were visually inspected and a threshold for framewise displacement (FD) was applied (maximum 40% of timepoint with FD>0.5), no subjects had to be excluded.
The R package influence.ME package was used to detect and remove influential cases based on Cook's distance: distance > 4 * mean distance (Pinho et al. 2015, Computational Statistics & Data Analysis).

Replication

The behavioral results of experiment 1 (enhanced motor performance during motor learning in the tTIS condition) were replicated in an additional cohort of older adults in experiment 2.

Randomization

All participants performed all stimulation conditions in a cross-over design.
The order of stimulation followed an a-priori defined pseudo-randomized sequence.

Blinding

The study was double-blinded.

Reporting for specific materials, systems and methods

We require information from authors about some types of materials, experimental systems and methods used in many studies. Here, indicate whether each material, system or method listed is relevant to your study. If you are not sure if a list item applies to your research, read the appropriate section before selecting a response.

Materials & experimental systems

n/a	Involved in the study
☒	☐ Antibodies
☒	☐ Eukaryotic cell lines
☒	☐ Palaeontology and archaeology
☒	☐ Animals and other organisms
☒	☐ Clinical data
☒	☐ Dual use research of concern
☒	☐ Plants

Methods

n/a	Involved in the study
☒	☐ ChIP-seq
☒	☐ Flow cytometry
☐	☒ MRI-based neuroimaging

Magnetic resonance imaging

Experimental design

Design type

Resting-state (rs) and task-based fMRI (block design)

Design specifications

In Experiment 1, 15 young healthy subjects performed 4 sessions: 2 rs-fMRI and 2 task-based fMRI sessions.
During the rs-fMRI sessions, functional images were acquired during three resting state sequences lasting 8 minutes each, namely, before (pre), during, and after (post) stimulation, while subjects fixated on a white cross on a black background. During the task-based fMRI sessions, the participants performed six 9 minute 30 second training blocks with an approximately 1 minute 30 second break between blocks. Each block included ten 30-second repetitions of the motor task with the respective stimulation condition, alternated with 30 seconds of rest (fixation cross).

Behavioral performance measures

We measured the number of correct key presses during each 30-second repetition of the task-based fMRI.
Values were corrected by dividing by the number of correct key presses performed during the baseline repetition (no stimulation).

Acquisition

Imaging type(s)

Functional and structural

Field strength

3T

Sequence & imaging parameters

Structural and functional images were acquired using a 3T MAGNETOM PRISMA scanner (Siemens, Erlangen, Germany). The 3D MPRAGE sequence was used to obtain T1-weighted images with the following parameters: TR=2.3 s; TE=2.96 ms; flip angle=9°; number of slices=192; voxel size=1×1×1 mm; and field of view (FOV)=256 mm; matrix size = 192 x 240 x 256; orientation = sagittal, phase encoding dir = A >> P. Anatomical T2 images were collected with the following parameters: TR=3 s; TE=409 ms; flip angle=120°; number of slices=208; voxel size=0.8×0.8×0.8 mm; and FOV=320 mm; matrix size = 208 x 320 x 320; orientation = sagittal, phase encoding dir = A >> P. Echo-planar imaging (EPI) sequences were used to obtain functional images with the following parameters: TR=1.25 s; TE=32 ms; flip angle=58°; number of slices=75; voxel size=2 × 2 × 2 mm; and FOV=112 mm; matrix size = 208 x 320 x 320; orientation = sagittal, phase encoding dir = A >> P.

Area of acquisition

Whole-brain

Diffusion MRI ☐ Used ☒ Not used

Preprocessing

Preprocessing software	Functional imaging data were analyzed using Statistical Parametric Mapping 12 (SPM12; https://www.fil.ion.ucl.ac.uk/spm/) implemented in MATLAB R2018a (Mathworks, Sherborn, MA, USA). All functional images underwent the same preprocessing, including the following steps: slice time correction, spatial realignment to the first image, normalization to the standard MNI space and smoothing with a 6 mm full-width half-maximal Gaussian kernel. T1 anatomical images were coregistered to the mean functional image and then segmented to produce the forward deformation field used to normalize the functional images, allowing bias-corrected gray and white matter images to be obtained. The framewise displacement was calculated for each run to control head movement. The nonnormalized and normalized images were visually inspected to ensure good preprocessing quality. The signal-to-noise ratio was also computed to control for possible tTIS-related artifacts.<br>For resting-state fMRI data, independent component analysis (ICA)-based artifact removal was performed on the preprocessed, smoothed images using the GIFT toolbox (https://trendscenter.org/software/gift/).<br>The recon-all function of the Freesurfer software was run, taking structural T1w and T2w images as inputs (https://surfer.nmr.mgh.harvard.edu/). For each individual subject, the Brainnetome atlas (BNA, https://atlas.brainnetome.org/bnatlas.html) parcellation was computed and specific ROIs were then co-registered to the functional images and normalized to the MNI space.
Normalization	SPM normalisation to MNI space, linear and non-linear transformation based on deformation fields obtained from segmentation in SPM.
Normalization template	MNI152 T1, 1mm
Noise and artifact removal	Visual check for co-registration and normalisation. Framewise displacement (FD) was computed and subjects showing more than 40% of time points of FD larger than 0.5mm. No subjects were excluded based on this criterion.
Volume censoring	We did not apply volume censoring.

Statistical modeling & inference

Model type and settings	Resting-state fMRI: Seed-based connectivity analyses were implemented at the single-subject level by extracting the average time series within the striatal mask defined in the BNA atlas and including this time series as a regressor in a general linear model with six head motion parameters (three displacement motions and three rotation motions) and normalized time series in the white matter and cerebrospinal fluid.<br>Task-based fMRI: A general linear model was used to estimate the signal amplitude at the single-subject level. Six head motion parameters (three displacement motions and three rotation motions) and the normalized time series in the white matter and corticospinal fluid were included as regressors.
Effect(s) tested	Resting-state fMRI:<br>- 1 sample t-test on the first-level contrast "tTIS versus HF control" and "HF control versus tTIS".<br>- Flexible factorial design was used to compute group-level statistics, including subject, stimulation and period as factors on the first-level contrasts (tTIS and HF control during pre, during and post-stimulation). In the matrix the stimulation effect (2 levels) was followed by the block effect (3 levels), then the interactions (tTIS and HF control for each period).<br>interaction contrasts:<br>0 0 0 0 0 1 0 -1 -1 0 1 0 0 0 0 0 0 0 0 0 0 0 0 0 0 0<br>0 0 0 0 0 -1 0 1 1 0 -1 0 0 0 0 0 0 0 0 0 0 0 0 0 0 0<br>Task-based fMRI:<br>- 1 sample t-test on the first-level contrast "HF control" for control activity during task<br>- Flexible factorial design was used to compute group-level statistics, including subject, stimulation and time as factors on the first-level contrasts (tTIS per block and HF control per block). In the matrix the stimulation effect (2 levels) was followed by the block effect (6 levels), then the interactions (tTIS with each block and HF control with each block).<br>tTIS versus HF control contrast: 1 -1 0 0 0 0 0 0 1/6 1/6 1/6 1/6 1/6 1/6 -1/6 -1/6 -1/6 -1/6 -1/6 -1/6 0 0 0 0 0 0 0 0 0 0 0 0 0 0<br>Linear increase for tTIS contrast: 0 0 -5 -3 -1 1 3 5 -5 -3 -1 1 3 5 0 0 0 0 0 0 0 0 0 0 0 0 0 0 0 0 0 0 0 0<br>Linear decrease for tTIS contrast: 0 0 5 3 1 -1 -3 -5 5 3 1 -1 -3 -5 0 0 0 0 0 0 0 0 0 0 0 0 0 0 0 0 0 0 0 0<br>Linear increase for HF control contrast: 0 0 -5 -3 -1 1 3 5 0 0 0 0 0 0 -5 -3 -1 1 3 5 0 0 0 0 0 0 0 0 0 0 0 0 0 0<br>Linear decrease for HF control contrast: 0 0 5 3 1 -1 -3 -5 0 0 0 0 0 0 5 3 1 -1 -3 -5 0 0 0 0 0 0 0 0 0 0 0 0 0 0

Specify type of analysis: ☐ Whole brain ☐ ROI-based ☒ Both

Anatomical location(s)	ROI based: An external radiologist manually drew striatal masks on each subject’s structural T1w image. After drawing the masks for caudate and putamen, anterior and posterior sub-parts were distinguished in respect to the location of the anterior commissure.<br>In SOM, control analysis were carried out in the following BNA atlas ROIs:<br>the left and right dorsal area A9/46d (15 and 16) and the left and right anterior superior temporal sulcus (aSTS) (87 and 88).
Statistic type for inference<br>(See Eklund et al. 2016)	Whole brain HF control activity: Voxel-wise uncorrected p=0.001 and cluster FDR corrected p=0.05<br>tTIS versus HF control: Voxel-wise uncorrected p=0.001 and cluster FDR corrected p=0.05<br>Linear increase or decrease within the right striatum: Voxel-wise uncorrected p=0.01 and uncorrected at the cluster level<br>Behavioral modulation: Voxel-wise uncorrected p=0.001 and cluster FDR corrected p=0.05
Correction	Whole brain HF control activity: Voxel-wise uncorrected p=0.001 and cluster FDR corrected p=0.05<br>tTIS versus HF control: Voxel-wise uncorrected p=0.001 and cluster FDR corrected p=0.05<br>Linear increase or decrease within the right striatum: Voxel-wise uncorrected p=0.01 and uncorrected at the cluster level

Behavioral modulation: Voxel-wise uncorrected p=0.001 and cluster FDR corrected p=0.05

Models & analysis

n/a	Involved in the study
☐	☒ Functional and/or effective connectivity
☒	☐ Graph analysis
☒	☐ Multivariate modeling or predictive analysis

Functional and/or effective connectivity

Seed-based rs-fMRI connectivity.
Generalized psychophysiological interaction for task-based design.
